# Comparative genomics of *Clostridioides difficile* toxinotypes identifies module-based toxin gene evolution

**DOI:** 10.1099/mgen.0.000449

**Published:** 2020-10-08

**Authors:** Sandra Janezic, Kate Dingle, Joseph Alvin, Tomaž Accetto, Xavier Didelot, Derrick W. Crook, D. Borden Lacy, Maja Rupnik

**Affiliations:** ^1^​ National Laboratory for Health, Environment and Food, Maribor, Slovenia; ^2^​ University of Maribor, Faculty of Medicine, Maribor, Slovenia; ^3^​ Oxford University, Nuffield Department of Clinical Medicine, John Radcliffe Hospital, Oxford, UK; ^4^​ Vanderbilt University School of Medicine, Nashville, TN, USA; ^5^​ Biotechnical Faculty, Animal Science Department, University of Ljubljana, Domzale, Slovenia; ^6^​ School of Life Sciences and Department of Statistics, University of Warwick, Coventry, UK; ^7^​ The Veterans Affairs Tennessee Valley Healthcare System, Nashville, TN, USA

**Keywords:** CdtLoc, *Clostridioides difficile*, evolution, PaLoc, toxin gene, toxinotypes

## Abstract

*
Clostridioides difficile* is a common cause of nosocomial diarrhoea. Toxins TcdA and TcdB are considered to be the main virulence factors and are encoded by the PaLoc region, while the binary toxin encoded in the CdtLoc region also contributes to pathogenicity. Variant toxinotypes reflect the genetic diversity of a key toxin-encoding 19 kb genetic element (the PaLoc). Here, we present analysis of a comprehensive collection of all known major *C. difficile* toxinotypes to address the evolutionary relationships of the toxin gene variants, the mechanisms underlying the origin and development of variability in toxin genes and the PaLoc, and the relationship between structure and function in TcdB variants. The structure of both toxin genes is modular, composed of interspersed blocks of sequences corresponding to functional domains and having different evolutionary histories, as shown by the distribution of mutations along the toxin genes and by incongruences of domain phylogenies compared to overall *C. difficile* cluster organization. In TcdB protein, four mutation patterns could be differentiated, which correlated very well with the type of TcdB cytopathic effect (CPE) on cultured cells. Mapping these mutations to the three-dimensional structure of the TcdB showed that the majority of the variation occurs in surface residues and that point mutation at residue 449 in alpha helix 16 differentiated strains with different types of CPE. In contrast to the PaLoc, phylogenetic trees of the CdtLoc were more consistent with the core genome phylogenies, but there were clues that CdtLoc can also be exchanged between strains.

## Data Summary

The sequence reads of the toxinotypes were deposited in the Sequence Read Archive (SRA accession: PRJNA605705). The authors confirm that all supporting data, code and protocols have been provided within the article or through supplementary data files.

**Impact Statement**

*
Clostridioides difficile* is the most important cause of nosocomial diarrhoea in industrialized countries and two toxins, A and B, are important for disease development. During evolution variant genes for toxins A and B were assembled by modules corresponding to the functional domains (catalytic, proteolytic, translocation, binding), which could represent the general feature of bacterial toxin origin. Toxin building blocks were exchanged within and between clades and some combinations are more common across the species than others. Additionally, the spectrum of variability presented here is important for the development of molecular tests for *C. difficile* diagnostics and for *C. difficile* vaccine development. The additional *C. difficile* toxin, binary toxin CDT, was also investigated but does not show such extensive variability as the PaLoc region.

## Introduction

*Clostridioides difficile* is a Gram-positive, sporogenic, anaerobic bacterium and is an important cause of healthcare- and community-associated intestinal infections in humans. The clinical manifestations of *C. difficile* infection (CDI) range from mild diarrhoea to pseudomembranous colitis, and the mortality rate is substantial [1, 2]. The main virulence factors are two exotoxins, toxin A (TcdA) and toxin B (TcdB), that belong to the family of large clostridial toxins (LCT). These toxins inactivate host Rho-GTPases by glucosylation, leading to disorganization of the cytoskeleton and cell death [3, 4]. In addition to TcdA and TcdB, some *C. difficile* strains produce a third, unrelated ADP-ribosylating toxin (CDT), which most likely contributes to pathogenesis, but the precise role is not yet fully understood [5, 6]. Strains that do not produce any of the three toxins are non-toxigenic and do not cause the disease.

TcdA and TcdB are encoded within the 19 kb well-studied genomic region called the pathogenicity locus (PaLoc). PaLoc also includes three accessory genes, positive (*tcd*R) and negative (*tcd*C) regulators of toxin expression and a gene coding for a putative holin-like protein (*tcd*E), responsible for secretion of the toxins [7, 8]. Binary toxin CDT is encoded in a distinct chromosomal region (Cdt locus; CdtLoc), which carries genes for catalytic (*cdt*A) and binding/translocation (*cdt*B) proteins and a regulatory protein (*cdt*R) [9]. CdtLoc is present in either a whole or a truncated version, while in strains lacking the CdtLoc, a unique 68 bp sequence is found inserted in this genomic location [9, 10].

Historically, genetic variation of the PaLoc has been assessed by toxinotyping, a PCR-RFLP method that distributes strains into 34 toxinotypes (I to XXXIV) based on differences in the PaLoc [11–13]. Different toxinotypes produce toxins that can vary in their biological effects and interactions with antibodies [4, 11]. Toxinotypes are therefore important from clinical, diagnostic, research and development perspectives. However, so far, only a few toxinotype representatives have been included in comparative genomic studies, performed either for epidemiological purposes [14–17] or for understanding virulence [18–20]. In addition, the evolution of the PaLoc was previously investigated with respect to the known population structure of the species, but in these reports not all known PaLoc variants, as defined by toxinotype, were included [21–24]. In the current study we present a comprehensive analysis of all major toxinotypes, elucidating the main features and possible driving forces in the origin and evolution of the PaLoc and more specifically its toxin genes. Additionally, we explore possible functional effects of amino acid substitutions by mapping to known toxin protein structures. Also included is a comparison of variability in two main toxin loci, PaLoc and CdtLoc.

## Methods

### Strains and genome sequencing

Genomes from 28 well-characterized reference toxinotypes (21 toxinotypes and subtypes) were included in the analysis (Table 1); 25 had full-length or truncated forms of *tcd*A, and of these, 19 strains produced toxin TcdA. In three toxinotypes, *tcd*A was completely absent. Twenty-six strains harboured full-length genes for TcdB and were all phenotypically B+, while two toxinotypes lacked the *tcd*B gene. Minor toxinotypes were not included in the analysis (I, II, XIII, XVIII-XX, XXVI, XXVII, XIX) as they arise spontaneously by recombination in CROP (repetitive parts of toxins) regions and do not represent evolutionarily stable genotypes. Toxinotypes XV, XVII, XXIII and XIV were previously reclassified into subtypes [13]. For further information on reference toxinotype strains, the reader is referred to Rupnik and Janezic [13].

**Table 1.** Characteristics of *C. difficile* toxinotypes included in the analysis (PCR ribotypes, toxin profiles, MLST-ST, clade and TcdA and CdtLoc variants)

Strain	Toxinotype	PCR ribotype	Toxin profile	MLST-ST	Clade	TcdA*	CdtLoc	NCBI RefSeq/ENA SRA accession no.
CD630	0	012	A+B+CDT−	54	1	Full	Truncated	NC_009089.1
597B	0/v	131	A+B+CDT+	122	1	Full	Full	PRJEB23450
SE844	IIIa	080	A+B+CDT+	192	2	Full	Full	This study
CD196	IIIb	027	A+B+CDT+	1	2	Full	Full	NC_013315.1
CH6230	IIIc	251	A+B+CDT+	123	2	Full	Full	This study
55 767	IV	023	A+B+CDT+	5	3	Full	Full	This study
SE881	V	045	A+B+CDT+	11	5	Full	Full	This study
51 377	VI	127	A+B+CDT+	11	5	Truncated†	Full	This study
57 267	VII	063	A+B+CDT+	193	5	Truncated†	Full	This study
1470	VIII	017	A−B+CDT−	37	4	Truncated‡	Absent	This study
51 680	IXa	019	A+B+CDT+	67	2	Full	Full	This study
8785	IXc	109	A+B+CDT+	196	2	Full	Full	This study
8864	Xa	59	A−B+CDT+	62	2	Truncated§	Full	This study
J9956	Xb	SLO 032	A−B+CDT+	194	2	Truncated§	Full	This study
IS58	XIa	033	A−B−CDT+	11	5	Truncated||	Full	This study
R11402	XIb	288	A−B−CDT+	11	5	Truncated||	Full	This study
IS25	XII	258	A+B+CDT−	58	1	Full	Truncated	This study
R10870	XIVa	111	A+B+CDT+	114	2	Full	Full	This study
R9385	XIVb	122	A+B+CDT+	116	2	Full	Full	This study
SUC36	XVI	078	A−B+CDT+	195	5	Truncated†	Full	This study
CH6223	XXI	SLO 035	A+B+CDT−	198	4	Full	Absent	This study
CD07-468	XXII	027	A+B+CDT+	197	2	Full	Full	This study
7325	XXV	027	A+B+CDT+	1	2	Truncated†	Full	This study
CD08-070	XXVIII	126	A+B+CDT+	11	5	Truncated†	Full	This study
ES130	XXX	SLO 101	A−B+CDT+	166	5	Absent	Full	PRJEB23450
WA151	XXXI	SLO 098	A−B+CDT+	167	5	Absent	Full	PRJEB23450
173070	XXXII	15	A−B+CDT−	200	C-II	Absent	Absent	This study
2402	XXXIII	SLO 086	A+B+CDT−	199	1	Full	Truncated	This study

*Adapted from [13].

†Deletion in the CROP domain.

‡Phenotype A− is due to nonsense mutation in the TcdA, not deletion in the CROP domain.

§Deletion in translocation and binding domain. Only part of glucosyl-transferase domain is present.

||Deletion in the glucosyl-transferase domain.

Genomes of 25 *C *. *difficile* toxinotypes were sequenced at the Wellcome Trust Centre for Human Genetics, using the methodology described previously [21]. The strain of toxinotype 0/v (ST-122) was sequenced using the Illumina MiSeq platform. Paired-end libraries were prepared with the Nextera XT sample preparation kit (Illumina) and sequenced using the MiSeq Reagent kit v2. *De novo* genome assemblies were created using Velvet [25]. The sequence reads of the toxinotypes were deposited in the Sequence Read Archive (SRA accession: PRJNA605705).

For toxinotypes 0 and IIIb, previously sequenced genomes, CD630 (NC_009089.1) [26] and CD196 (NC_013315.1) [27], were used.

## Phylogenetic analysis

Genomes of 41 strains, representative of the diversity of the *C. difficile* population, from 6 clades (1, 2, 3, 4, 5 and C-I) [21] and 28 reference toxinotypes, were chosen for global phylogenetic analysis. The distribution of toxinotypes within the *C. difficile* population was assessed by construction of a neighbour-joining tree based on concatenated nucleotide sequences (total length of 1.26 Mb). A phylogenetic tree was constructed using FigTree software (http://tree.bio.ed.ac.uk/software/figtree/).

For *in silico* multilocus sequence typing (MLST), alleles were extracted from whole-genome sequences using BIGSdb [28]. Designations of alleles and sequence types (STs) were obtained by querying the PubMLST database. Newly identified alleles and sequence types were submitted to the PubMLST database (http://pubmlst.org/cdifficile).

For phylogenetic analysis of genes within the PaLoc and CdtLoc, sequences were extracted from genomes using BIGSdb. Artemis was used to browse and annotate the genes of interest, and blast searches were used to confirm the identity of the genes. All sequences were aligned with clustal Omega [29], and maximum-likelihood trees were constructed in mega 6 [30].

### Mutation distribution analysis of the PaLoc and TcdB

For comparative analysis of the genetic variation across the PaLoc, sequences were first aligned to the PaLoc of *C. difficile* reference genome CD630 and the number of substitutions was counted using a 50–100 nucleotide sliding window. The distribution of substitutions in TcdB was assessed by counting the substitutions in a 100 amino acid sliding widow after aligning the sequence to CD630 TcdB.

### Phylogenetic network and pairwise homoplasy test

The neighbour-net method within SplitsTree version 4 (http://www.splitstree.org/) was used to detect phylogenetic evidence of recombination, and for statistical verification of recombination, the pairwise homoplasy test (PHI) was applied [31].

### Structural analysis of different TcdB variants

DNA sequences of TcdB were translated to their corresponding protein sequences. The amino acid sequences were aligned using the clustal Kalign algorithm. These sequences were submitted to the SWISS-MODEL server and the TcdA 1–1832 crystal structure (PDB ID: 4R04) was used as the model template [32]. Figures were generated using the built-in functions of PyMol (v1.8, Schrödinger, LLC). Residues strictly conserved across all five clades were coloured red. This selection was inverted and coloured blue to represent residues that varied within the datasets. Vacuum electrostatics were generated using PyMol’s built-in function, set to represent the charge at one solvent radius.

## Results

### Distribution of toxinotypes in the *C. difficile* population structure

**Fig. 1.**
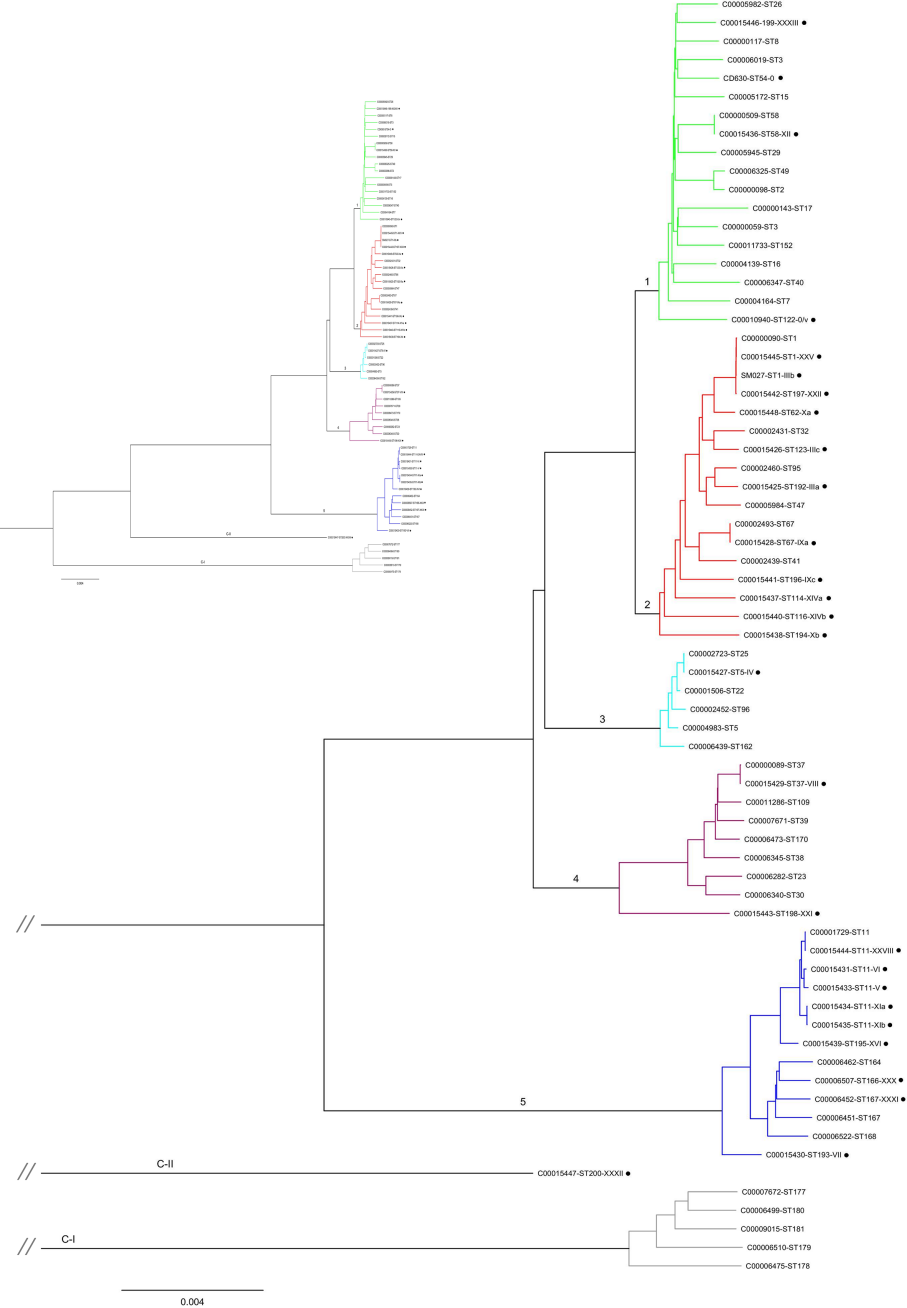
Phylogenetic relationships of *C. difficile* based on whole-genome sequences. A neighbour-joining tree using 1434 shared genes, with a concatenated length of 1.26 Mb constructed from a total of 69 genomes (28 toxinotypes plus 41 strains covering all 6 clades). Toxinotypes are marked with black dots.

The distribution of toxinotypes, reflecting PaLoc variants, within the *C. difficile* population was determined by the construction of a phylogenetic tree based on a core genome of 69 *C *. *difficile* isolates (28 toxinotypes included in this study and 41 representatives of the 6 clades, as described previously [21] (Fig. 1). Reference toxinotype strains occurred in all of the main clades. Clades 2 and 5 contained the greatest numbers of different toxinotypes, with 11 and 9 PaLoc variants detected, respectively. The number of reference toxinotypes was smaller in other clades, with four toxinotypes grouped in clade 1 and two toxinotypes in clade 4. In clade 3 only toxinotype IV PaLoc was present. A member of cryptic clade C-II included a single toxinotype (toxinotype XXXII, ST 200, A−B+CDT−).

### Phylogenetic relationships of the toxin variants and other PaLoc regions

**Fig. 2.**
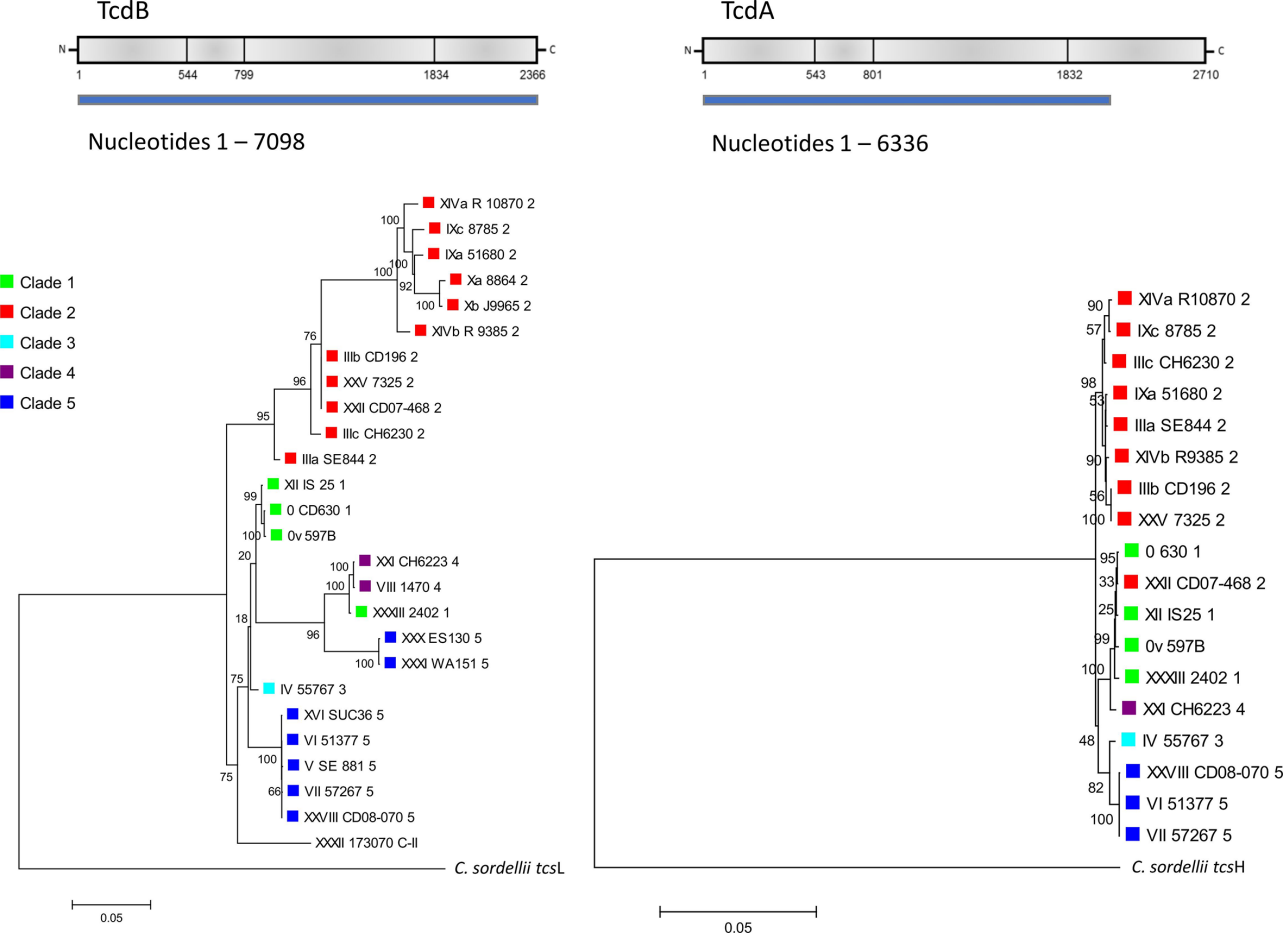
Maximum-likelihood trees based on nucleotide sequences of *tcd*B and *tcd*A. For *tcd*A we only included toxinotypes with A+ phenotype in the analysis and because of deletions present in the binding domain of *tcd*A, only the first 6336 nucleotide sites were analysed. Toxinotype V (SE881) is not included because of low sequencing quality. *C. sordellii tcs*L and *tcs*H were used to root the tree. Coloured shapes indicate the clade (Fig. 1).

Phylogenies based on *tcd*A and *tcd*B alone vary in congruence with whole-genome phylogenies. Some of the branches in toxin gene cladograms correlate entirely with overall genome phylogeny based on clade definition, while other branches include representatives from several clades, and this is observed for both toxins (Fig. 2). Specifically, the *tcd*B of toxinotype XXXIII from clade 1 grouped with the two toxinotypes from clade 4 (i.e. XXI and VIII), and the *tcd*A of toxinotype XXI and XXII from clades 4 and 2, respectively, grouped with toxinotypes from clade 1 (Fig. 2).

**Fig. 3.**
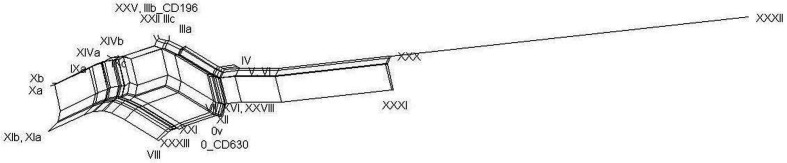
Neighbour-net generated for the PaLoc region. Box-like relationships among the toxinotypes instead of a bifurcating tree indicate the presence of recombination.

**Fig. 4.**
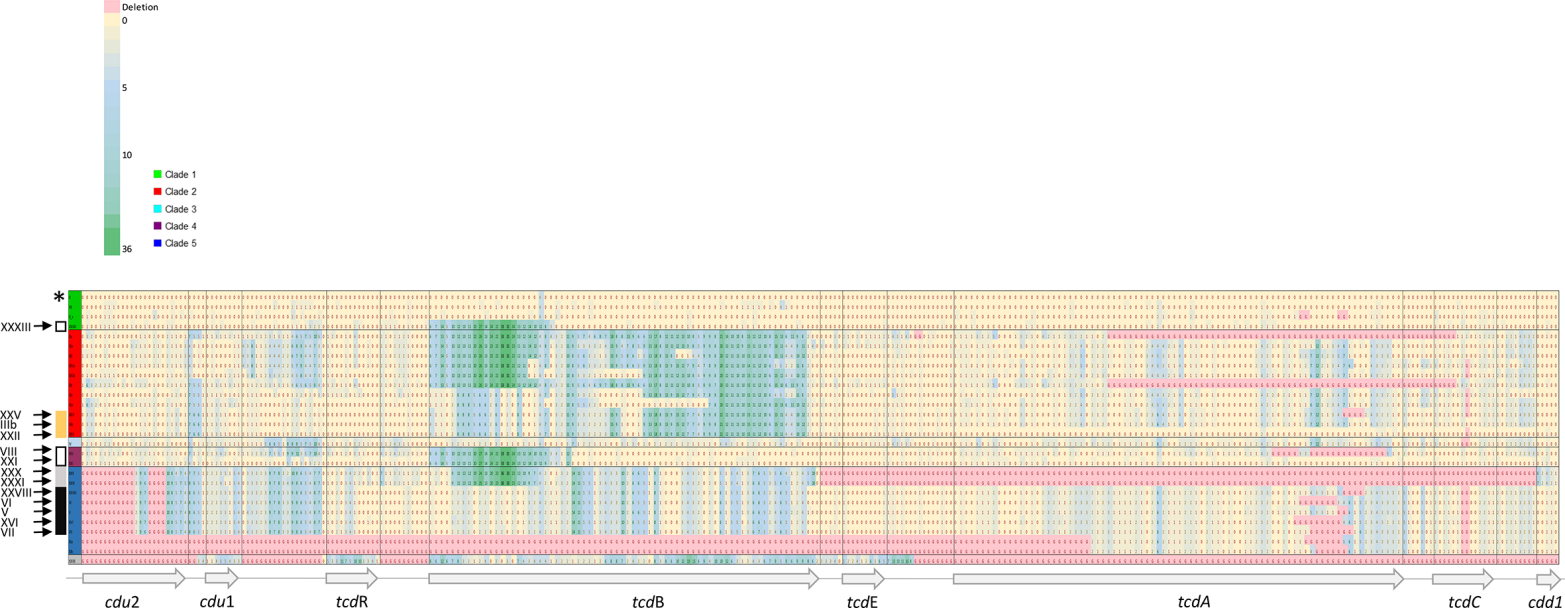
Density and patterns of mutation distribution along the PaLoc. Heatmap of SNP density in 50 or 100 bp windows along the PaLoc. Nucleotide sequences of the entire PaLoc were compared to a reference strain CD630 (toxinotype 0). The heatmap is sorted by clades: green, clade 1; red, clade 2; light blue, clade 3; purple, clade 4; dark blue, clade 5. Gene *tcd*B is the most variable part of the PaLoc, considering only SNPs. * Coloured boxes denote toxinotypes, whose phylogenetic relationships change along the PaLoc region (see Table S1).

The lack of congruence between the toxin gene and whole-genome phylogenies prompted us to explore this in more detail. First, we constructed a neighbour-net network on the concatenated alignment of the entire PaLoc region. A box-like topology of the network confirmed conflicting phylogenetic signals, which indicates possible recombination events among the toxinotypes (Fig. 3). The PHI test also strongly supported the occurrence of recombination (*P* <0.001, based on 1699 informative sites). These recombination ‘hot regions’ within the PaLoc (the locations where recombination occurs frequently) were visualized by mapping the mutation distribution along the entire PaLoc and a flanking regions (coding and intergenic regions). Sequence blocks with similar mutation patterns that are likely exchanged between toxinotypes occur along the entire PaLoc (Fig. 4, Table S1, available in the online version of this article.).

Additionally, we constructed phylogenies for individual genes and the intergenic regions of the PaLoc alignment, which demonstrated topological differences between the trees (Fig. S1). Both approaches clearly show the mosaicism of the PaLoc, in particular in *tcd*B. For instance, the PaLoc sequence in toxinotypes from clade 5 (grey and black boxes in Figs 4 and S1) is almost the same up to the *tcd*R*–tcd*B intergenic region. Subsequently, a distinct *tcd*B-GT (glucosyl-transferase) domain is found in toxinotype XXX and XXXI compared to other five clade 5 toxinotypes (V, VI, VII, VXI, XXVII). In the translocation and receptor-binding domains their sequences again become very similar. In toxinotypes IIIb, XXII and XXV from clade 2 (orange boxes in Fig. S1) the PaLoc is almost identical up to *tcd*A, where XXII becomes similar to toxinotypes from clade 1. Toxinotypes XXXIII (clade 1), VIII and XXI (clade 4) (black framed boxes), which exhibit rather variable PaLoc phylogenies, possess an identical *tcd*B-GT domain (up to 1800 bp) and their other *tcd*B domains are also rather similar.

### Variability of toxin genes and proteins and other PaLoc regions

The analysis of nucleotide and amino acid sequences demonstrated that *tcd*B/TcdB is more variable in terms of substitution accumulation and *tcd*A/TcdA is more conserved (Figs 2 and 4). *Tcd*B sequences show more deeply rooted distribution into groups, while for *tcd*A only three groups with smaller genetic distances are detected (Fig. 2). In *tcd*B the total number of polymorphic sites was 1228/7098 (17.3 %) in the nucleotide sequence and 500/2366 (21.1 %) in the amino acid sequence, while in the *tcd*A gene only 158/6338 (2.5 %) of nucleotide sites and 49/2111 (2.3 %) amino acid sites were variable. In *tcd*A/TcdA, only the first 6337 nucleotide or 2111 amino acid sites were included in the analysis, due to deletions in repetitive regions, which are typically present.

Among the three accessory genes, *tcd*R seem to be most variable, based on the number of polymorphic sites (99/555; 17.8 %). However, only 1 of 28 strains (toxinotype XXXII) made the principal contribution to this variability seen in *tcd*R. Excluding this toxinotype from the *tcd*R analysis, the proportion of polymorphic sites is more comparable between all accessory PaLoc genes, 8.5, 4.7 and 7.6 % for *tcd*E, *tcd*C and *tcd*R, respectively.

### Modular distributions of amino acid substitutions in TcdB and association with two distinct types of cytopathic effect (CPE)

**Fig. 5.**
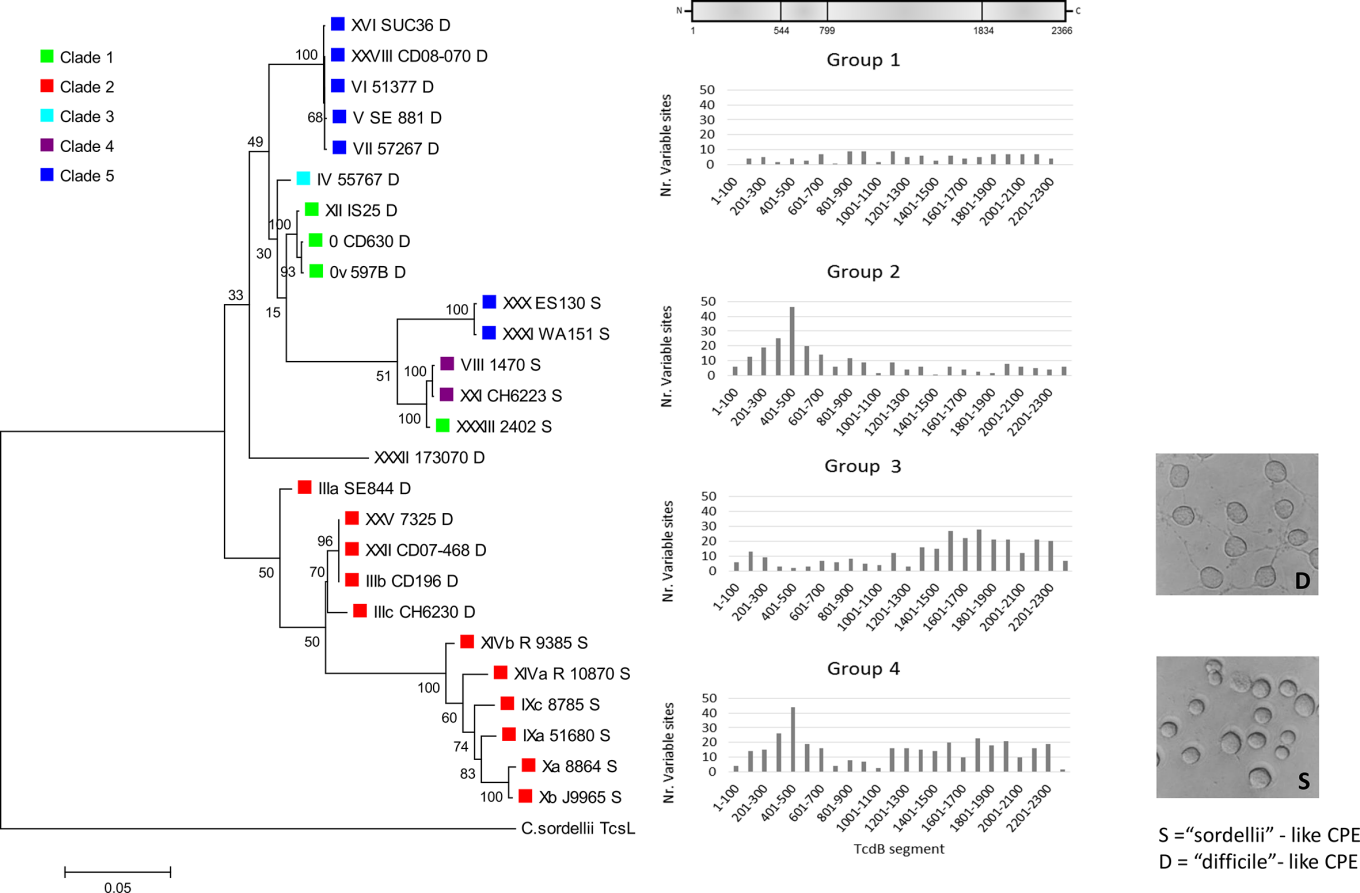
Four patterns of point mutation distribution along TcdB proteins and their association with the type of cytopathic effect on cell lines. Amino acid sequences of the full-length TcdB were compared with the TcdB of a reference strain CD630 (toxinotype 0) [26]. Coloured shapes indicate the clade (Fig. 1). S or D after the strain name indicates the type of the CPE (D is ‘difficile’- and S is ‘sordellii’-like CPE). Strains from groups 1 and 3 have difficile-like CPE and strains from groups 2 and 4 have sordellii-like CPE.

Four groups of substitution patterns were defined according to the distribution of amino acid differences along the TcdB protein (Fig. 5): (1) amino acid substitutions were rare and equally distributed along the protein, (2) substitutions were mainly found in catalytic/proteinase domain, (3) substitutions were mainly found in the receptor-binding/translocation domains and (4) substitutions were mainly present in the receptor-binding/translocation and catalytic/proteinase domains.

**Fig. 6.**
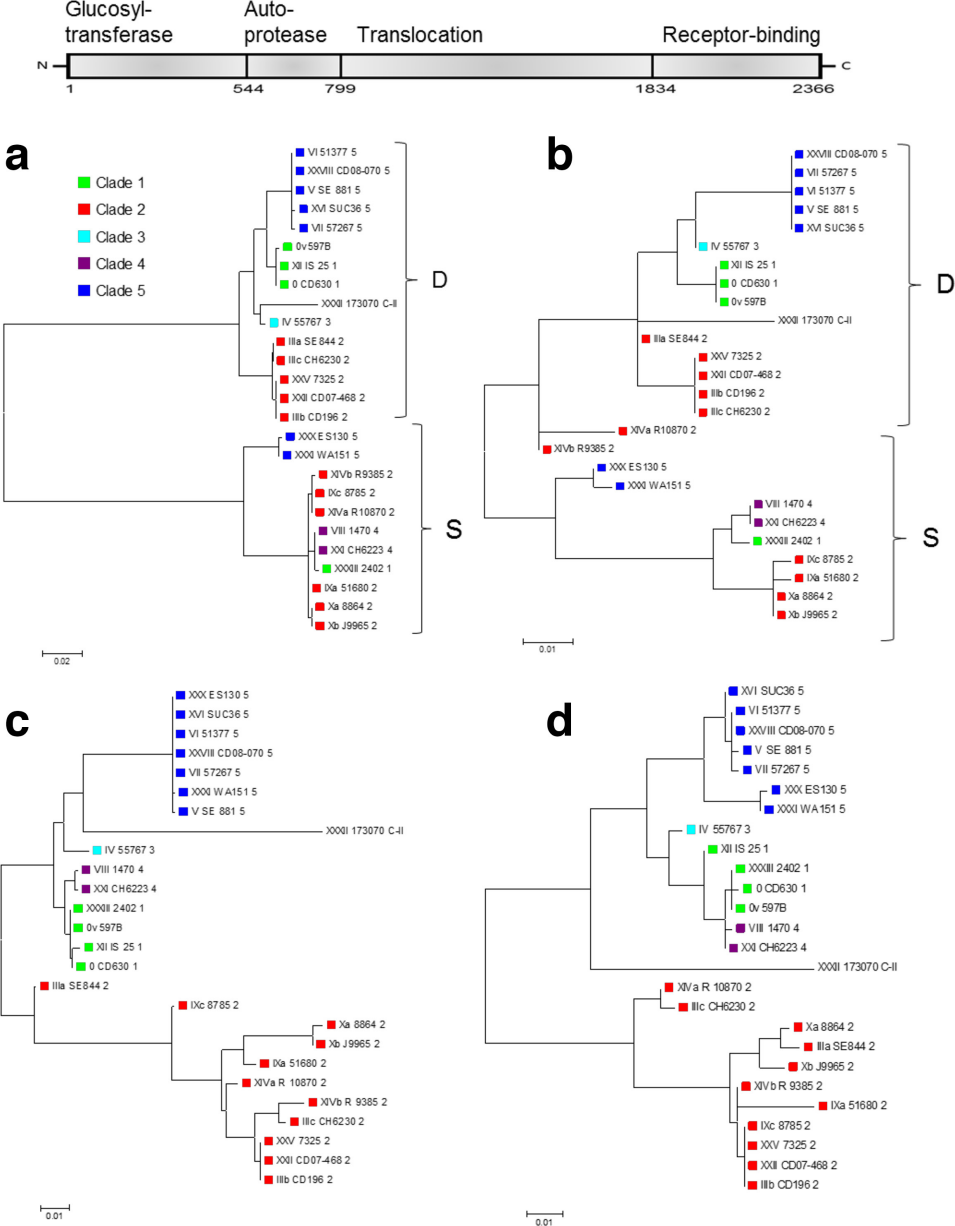
Phylogenies of the TcdB constructed from each of the four functional domains. Phylogenetic relationships based on glucosyl-transferase domain (a), autoprotease domain (b), translocation domain (c) and CROP domain (d). Mainly in the GTD (a) and autoprotease domain (b), two groups are differentiated, which correlates well with the type of CPE (S, sordellii-like; D, difficile-like). The groups are also not congruent with the phylogeny based on whole-genome sequencing (see Fig. 1). Coloured shapes indicate the clade (Fig. 1).

These amino acid substitution patterns coincided well with the type of CPE of culture supernatant on cells. In general, most *C. difficile* strains cause the difficile type of CPE, where cells round up but still show long protrusions. TcsL produced by *Clostridium sordellii *, related to TcdB, causes different CPE, where cells round up without any protrusions [11]. It is known that both types of CPE can be detected with variant *C. difficile* strains or isolated TcdB, difficile-type or sordellii-like CPE. We show here that sordellii-like CPE correlates well with TcdB variants with high substitution density in the catalytic domain (groups 2 and 4), while difficile-type CPE corresponds to groups 1 and 3 with less variance in the catalytic domain (Fig. 5). Further, cladograms of the catalytic (GTD) and autoprotease domain of TcdB fit better to the grouping of toxins according to CPE, but not so much their clade identity (Fig. 6a, b). However, the relationship between toxinotypes changes along the TcdB, up to the translocation and receptor-binding domains, when they become congruent with the genome-wide phylogeny (Fig. 6c, d).

### Structural analysis of different TcdB variants

**Fig. 7.**
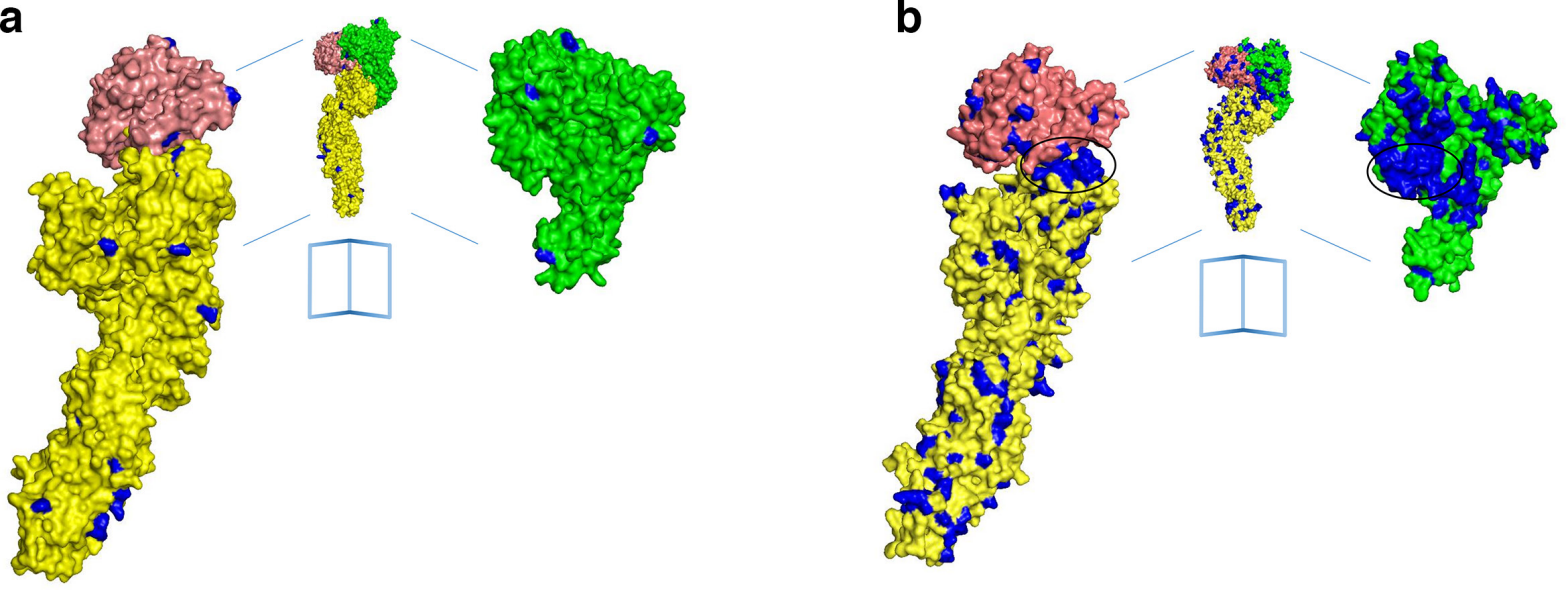
Mapping TcdA and TcdB sequence variation on crystal structures of TcdA. (a) Open book figure of a TcdA model (residues 1–1832) with variable residues shown in blue. Conserved residues are shown in green (GTD), salmon (APD) and yellow (PFD). (b) Open book figure of a TcdB model (residues 1–1832) with variable residues shown in blue. Conserved residues are shown in green (GTD), salmon (APD) and yellow (PFD). GTD, glucosyl-transferase domain; APD, autoprotease domain; PFD, pore-forming/translocation domain.

The crystal structure of residues 1–1832 of TcdA [3] provided an opportunity to examine how the sequence variation within TcdA and TcdB maps to the three-dimensional structure of the glucosyl-transferase domain (GTD), autoprotease domain (APD) and pore-forming/translocation domain (PFD). Aligning either TcdA (Fig. 7a) or TcdB sequences (Fig. 7b) to the structure reveals that the majority of the variation occurs in surface residues, consistent with the expectation that TcdA and TcdB adopt similar structures and that residues important for the structural integrity of the protein will be conserved between the toxins and across all clade families. However, there is one buried region of variation that can be observed in the examination of variation across TcdB sequences (Fig. 7b). This region of variation is located in the face of the TcdB GTD that is proposed to interact with GTPase and in the corresponding region of the APD that interacts with this site in the context of the holotoxin (Fig. 7b). It is thought that this region of variation influences the CPE [33].

**Fig. 8.**
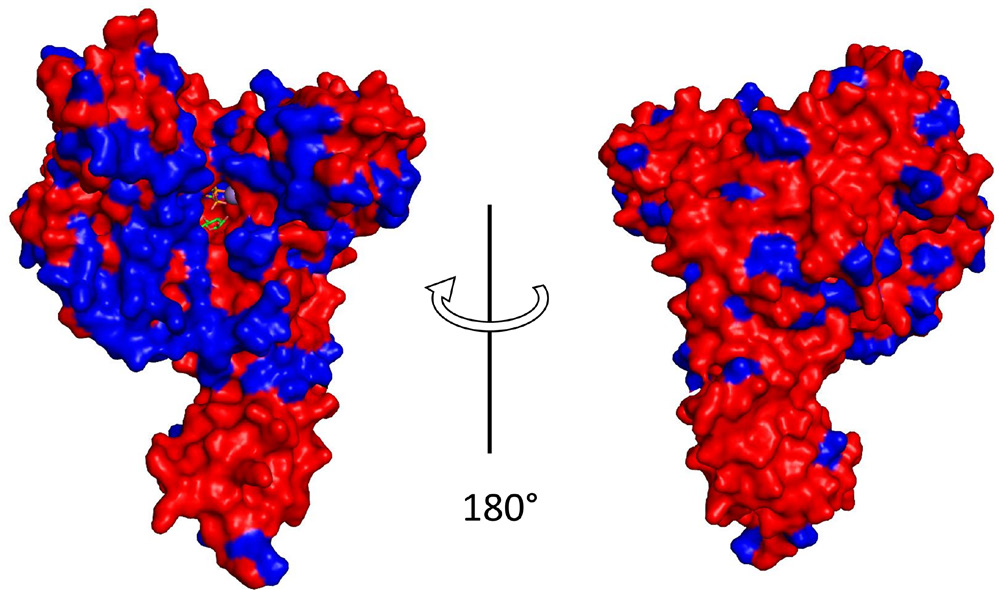
TcdB-GTD sequence variation across the TcdB sequence family. The TcdB GTD structure (PDB 2BVL) is depicted as a surface rendering with strictly conserved residues indicated in red. The majority of amino acid variations (blue) occur on the face of TcdB-GTD that is thought to interact with GTPase substrate.

**Fig. 9.**
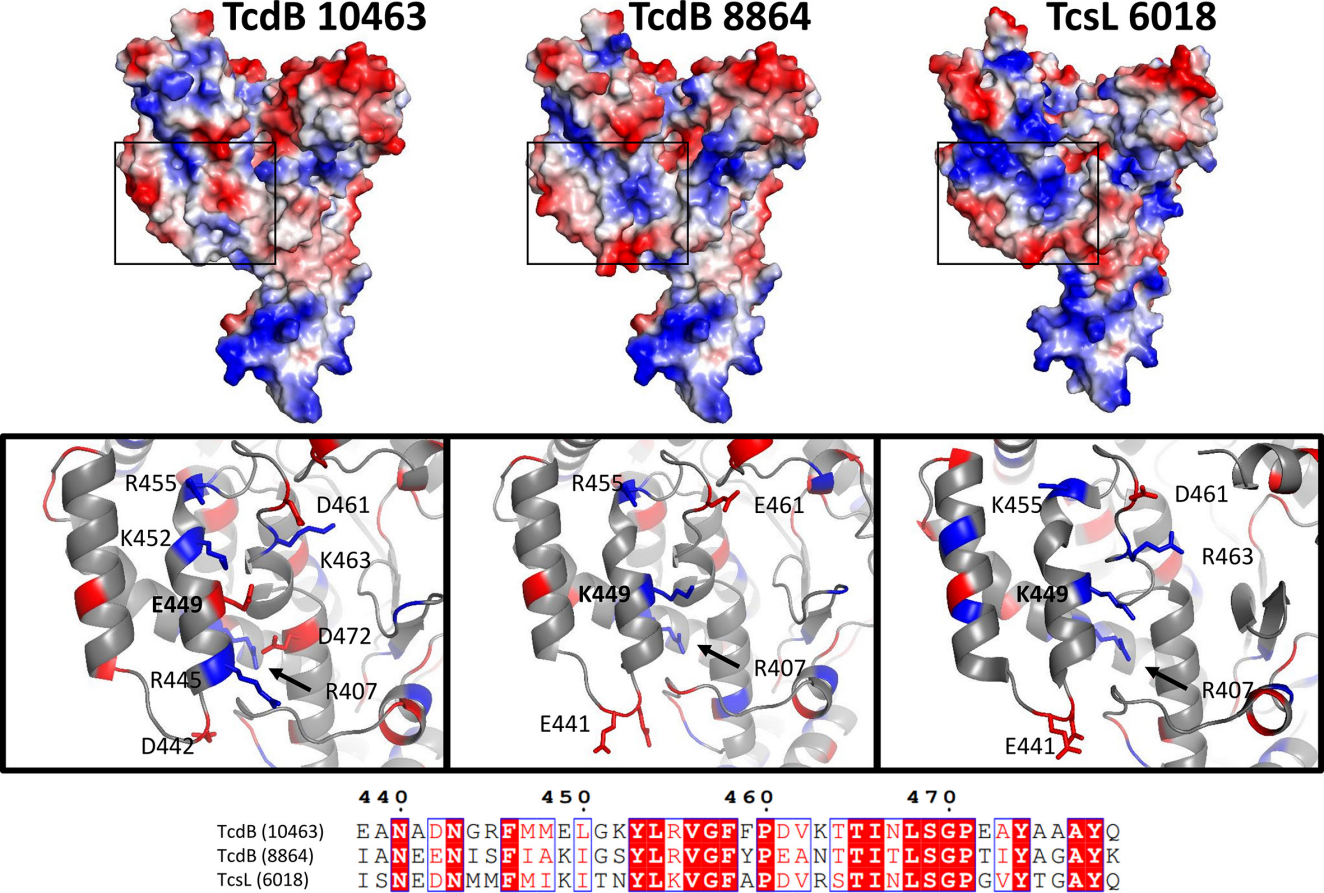
Electrostatic renderings of glucosyltransferase domains for TcdB and TcsL. Arrows indicate regions of similarity between TcdB-GTD strain VPI10463 (left), TcdB-GTD strain 8864 (centre) and TcsL-GTD (right). Boxed selections highlight the charged sidechains of helices 16 and 17 labelled according to the sequence alignment. Positive and negatively charged amino acids are coloured blue and red, respectively.

To understand the evolutionary processes behind CPE variation, the distribution of variable amino acid sites in TcdB from toxinotypes VIII (strain 1470) and Xa (strain 8864), representative of groups 2 and 4, respectively (Fig. 5), have been explored in more detail. Interestingly, the majority of variable sites within the GTD were located on the concave face, which is thought to bind and facilitate the transfer of glucose from UDP-glucose to target GTPases (Fig. 8). By modelling the TcdB-GTD from strain 8864, we compared the electrostatic predictions to the crystal structures of TcdB VPI10463 (PDB 2BVL) [34] from *C. difficile* and TcsL 6018 (PDB 2VK9) from *C. sordellii* (Fig. 9). The boxed areas represent the surface of alpha helices 16–17, which are more positively charged in both TcsL 6018 and TcdB 8864. This similarity between TcdB 8864 and TcsL 6018 is located at residue 449 in alpha helix 16 (Fig. 9). All strains within groups 2 and 4 (sordellii-like CPE) have lysine at this position, compared to groups 1 and 3, which have glutamate.

### Variability of toxin genes within the CdtLoc

**Fig. 10.**
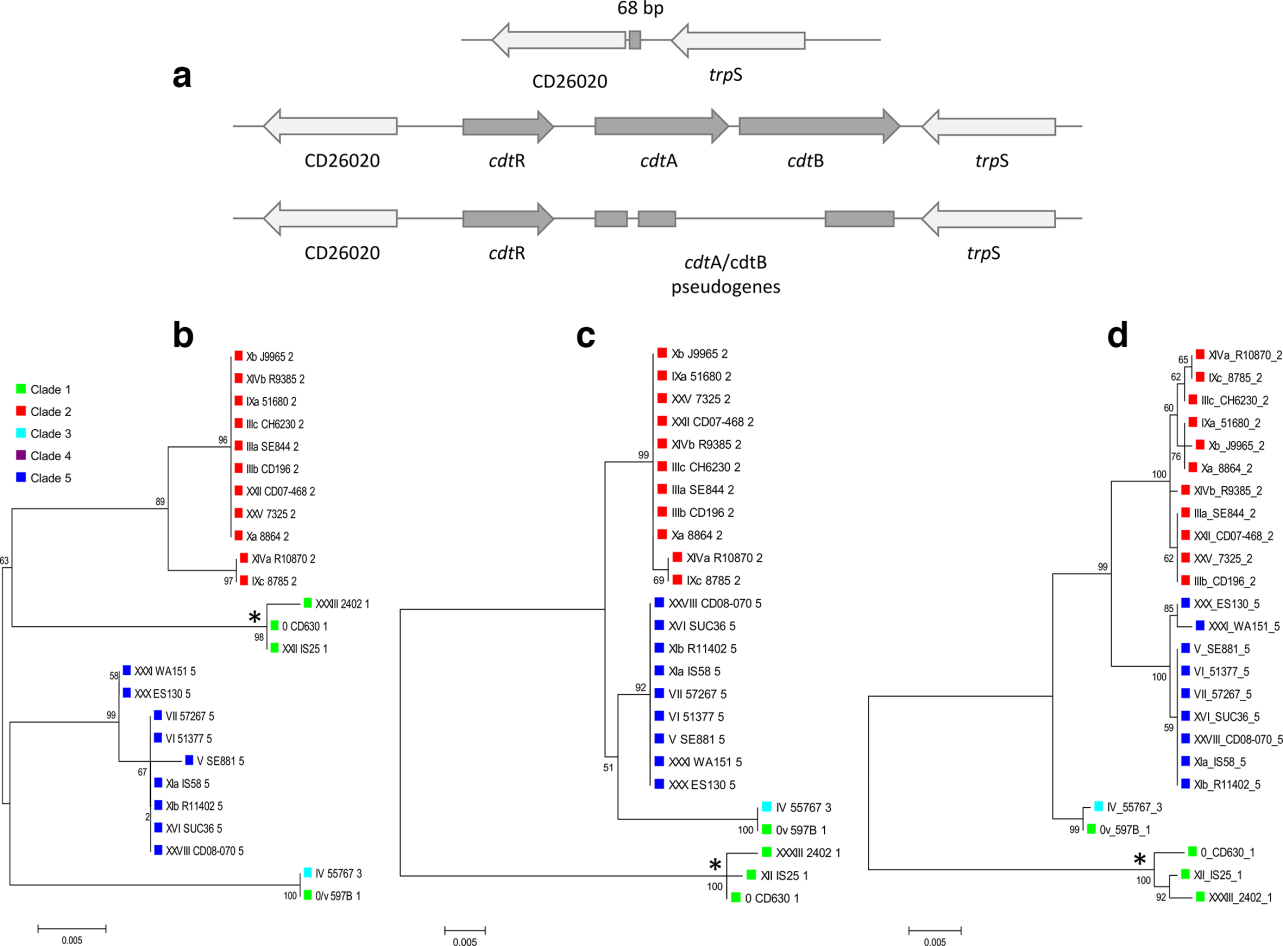
Phylogenetic relationships of CdtLoc genes and genetic organization of CdtLoc and its flanking genes. (a) Genomic organization in a CDT-negative strain where a unique 68 bp sequence is present, replacing the CdtLoc, CDT-positive strain and CDT-negative strain with a truncated form of the CdtLoc. Phylogenies of the CdtLoc genes (b) *cdt*R, (c) *cdt*A and (d) *cdt*B, which encode a regulatory protein, catalytic and binding/translocation domain, respectively. *Gene *cdt*R and cdtA/B pseudogenes from toxinotypes with a truncated form of the CdtLoc.

Annotation of the CdtLoc demonstrated that this locus was either present in full length or truncated forms or absent (Fig. 10a–c). The full-length CdtLoc was present in all strains from clades 2, 3 and 5. In clade 1, full-length (found in toxinotype 0/v) or truncated (found in toxinotypes 0, XII and XXXII) forms of CdtLoc were found. In clades 4 and C-II, CdtLoc was completely absent.

In contrast to the PaLoc genes, phylogenetic trees of all three genes within the CdtLoc demonstrated similar groupings to the core genome phylogenies, except the toxinotype 0/v strain (from clade 1), which grouped with the toxinotype IV strain (belonging to clade 4) in all three CdtLoc genes (Fig. 10). All CdtLoc genes from the truncated loci were clearly distinct from the full-length CdtLoc (Fig. 10b–d). Low variability was observed for both toxin genes, with only 3.0 % of variable nucleotide sites in full-length *cdt*A (41/1389) and *cdt*B (80/2634).

### Insertion sites of the PaLoc and CdtLoc

Annotations of the genomic region containing the PaLoc demonstrated that in all toxinotypes but one, the PaLoc was located at the same chromosomal site, namely between the *cdd1* and *cdu1* genes, as described previously [7]. The only exception is toxinotype XXXII, where PaLoc was found inserted at a different genomic location as described previously [35].

Analysis of CdtLoc and its flanking region demonstrated that in all the genomes in which it occurred, the locus was found to be stably integrated in an identical genomic location, between the genes CD26020 (5′-end) and *trp*S gene (3′-end) (Fig. 10a).

## Discussion

Although large genomic studies readily report variant forms of toxin genes, in *C. difficile* is toxinotyping the method of choice to systematically classify the diversity in genes for TcdA and TcdB. Currently 34 toxinotypes have been described and are distributed into major and minor toxinotypes. Minor toxinotypes only reflect recombination events in repetitive regions of *tcd*A and are not specifically linked with certain PCR ribotypes or MLST types. However, major toxinotypes show deletions and/or single-nucleotide variants (SNVs) along entire toxin genes, are genetically stable and mostly correlate well with certain PCR ribotypes. For this reason, only the major toxinotypes were included in this study. Including a single representative per toxinotype is one of the limitations of this study, but they do represent a well-characterized collection of variant strains [11].

### Evolution of *C. difficile* toxin genes – combinations of functional modules

The bimodal distribution of SNVs in *tcdB* and amino acid substitutions in TcdB indicates that this toxin is composed of two main building blocks (Fig. 5), the N terminal part comprising amino acids 1 to 800 covering the catalytic and autoprotease domain and the C terminal part of the toxin with translocation and CROP domains. This agrees with early observations of different homologies and suggests that TcdB evolved from multiple glycosyltransferase ancestors. The catalytic domains of TcdB and other LCTs are similar to proteins involved in the synthesis of capsular polysaccharides, adhesion molecules and yeast glycosyltransferases (OCH1). Repetitive parts of toxins (CROP domain) are similar to glycosyltransferases from oral streptococci and to lytic enzymes from *Streptococcus pneumoniae *; the common feature being saccharide, glucans or choline binding [36]. The analysis of toxin gene sequences of different toxinotypes shows that modules constituting the toxins could be even smaller. The phylogenies of all four functional domains of TcdB suggest that each functional domain could be an individual module. Moreover, each functional module has a unique evolutionary history. The glucosyltransferase and autoprotease domains are modules that seem to be most actively exchanged between variant forms. Evidence of recombination was observed most often in clade 2 strains, catalytic/autoprotease and translocation/binding domains being linked in different combinations (Figs 4 and 6).

Although there are several variant forms of the glucosyltransferase domain, they can be grouped functionally into two main clusters according to the form of CPE they cause (Fig. 5). Differences in cell morphology after treatment with different variant toxins are based on differences in the affected GTPases [37]. This was recognized in early studies characterizing the variant toxins of toxinotypes VIII, X and XIV [38–40]. In addition to Rho and Rac they also modify Rap, Ral and R-Ras, which makes them similar to *C. sordellii* TcsL. Mapping of amino acid substitutions in our study has shown that most of them are indeed located in the regions responsible for GTPase selection. Moreover, specific changes were defined that differentiated strains with different CPE.

Interestingly, the glucosyltransferase domain resulting in sordellii-like CPE was present in four clades (1, 2, 4 and 5). Clades 1, 2 and 5 have toxins with both difficile- and sordellii-like catalytic domains. Based on the phylogeny of the catalytic region, it could be concluded that the sordellii-like catalytic domain was introduced to *C. difficile* at least twice, once to clade 5 and once to clade 2. In clade 2 there were several exchanges within the clade 2 and between clades (to clades 1 and 4). It remains to be clarified why the translocation/receptor-binding domains are clade-associated and glucosyltransferase and autoprotease domains are readily exchanged between strains.

TcdA does not show such a clear bimodal SNV distribution as TcdB. The results presented here on the sequence level confirm the previous observations that TcdB is much more heterogeneous than TcdA. The reasons for this difference remain unknown.

### PaLoc evolution is also shaped by module reorganization

Similar to the toxin genes *tcd*A and *tcd*B, the entire PaLoc appears to have a modular structure. The variability in the PaLoc region is a result of individual nucleotide substitutions and recombination events. Phylogenetic trees (Fig. S1) and the SNV distribution density (Fig. 4) revealed interspersed blocks of sequences with different evolutionary histories, demonstrating that recombination (within and between clades) has played an important role in the evolution of the PaLoc variants (Figs 2, 4 and S1). However, in *C. difficile* recombination seems to be frequent enough to be detectable but not frequent enough to destroy all clonal signals.

Other PaLoc regions lacking congruence with the ‘core’ genome phylogeny (defined using whole-genome sequencing) occur in the nontoxin-encoding PaLoc genes and in intergenic regions between PaLoc genes. This indicates that different parts of PaLoc could act as modules that can be mobilized and exchanged. Recombination does require two copies of the genes to be present and this situation had not yet been proven in *C. difficile* strains. But it was shown that PaLoc could be transferred from a toxinogenic strain to a nontoxinogenic strain [41] and monotoxin variant forms of PaLoc in divergent strains were shown to be plasmid-associated [20]. Since signals of recombination were also found in regions adjacent to the PaLoc and in a previous study in the 16 S–23S rRNA intergenic spacer region (ISR) [42], one could speculate that the whole *C. difficile* genome is actually composed of recombined segments derived from different strains.

Previous evolutionary studies of the PaLoc demonstrated that each clade acquired a specific PaLoc variant independently, followed by subsequent exchanges and losses [21]. Our results have confirmed this and in addition shown that clades 2 and 5 are more likely to gain or evolve PaLoc variants than others. In particular, in clade 2 the PaLoc variants are very heterogeneous and show a large number of different evolutionary events (Figs 4 and 6).

### CdtLoc is more conserved than PaLoc and shows potential mobility

CdtLoc and PaLoc are two separate loci coding for different toxins. However, there is a pattern to their presence or absence and, even though toxintoypes do not have a common ancestor, it seems that the presence of CdtLoc is associated with PaLoc variants, while its absence is associated with strains with non-variant PaLoc. Full-length CdtLoc and CDT production are mainly observed in strains that have a significantly altered PaLoc [11]. Non-variant strains or strains with minor changes in the PaLoc (found mainly in clade 1 and also in clade 4) have a common truncation in the CdtLoc resulting in a CDT-negative phenotype [10, 43].

Not many studies have reported on the variability of CdtLoc. Metcalf and Weese [44] analysed the CdtLoc of 10 isolates belonging to four different toxinotypes (III, IV,V and IX; in this particular study the clade information is not given, but these toxinotypes are associated with clades 2, 3 and 5). In line with their study, low variability and no major differences in the number of polymorphic sites between *cdt*A and *cdt*B were detected in our set of strains. We observed that two phylogenetically distinct strains (based on core genome), which also differed in their toxinotype (PaLoc), shared identical Cdt loci, suggesting lateral CdtLoc exchange between different genomes (Fig. 10). Putative mobility of the CdtLoc via bacteriophages was recently suggested [45]. Even if CdtLoc is mobile, the question remains as to how it is transferred across strains separated by a wide genetic distance, i.e. from two distinct clades, and why it is only retained in strains with variant PaLoc.

In summary, the toxin genes *tcd*A and *tcd*B, as well as the entire PaLoc, have a modular structure, with each module having a unique mutation distribution. The main modules seem to correspond to the functional domains of TcdB, but could also be smaller or larger. Our results suggest that modules can be exchanged either within or between clades. Recombination hot spots are adjacent to the PaLoc, between the *tcdA* and *tcdB* toxin genes and between the catalytic/protease domain and the rest of the toxin(s). CdtLoc is much more conserved than PaLoc, but there are clues that exchange of CdtLoc has occurred between strains.

## Supplementary Data

Supplementary material 1
