# Supplementary material for: Comparative genomics of Clostridioides difficile toxinotypes identifies module-based toxin gene evolution
Source: Microb Genom. 2020 Oct 8;6(10):mgen000449. doi: 10.1099/mgen.0.000449 (PMC7660249; doi:10.1099/mgen.0.000449)
Supplement: Supplementary material 1 [file mgen-6-449-s001.pdf]

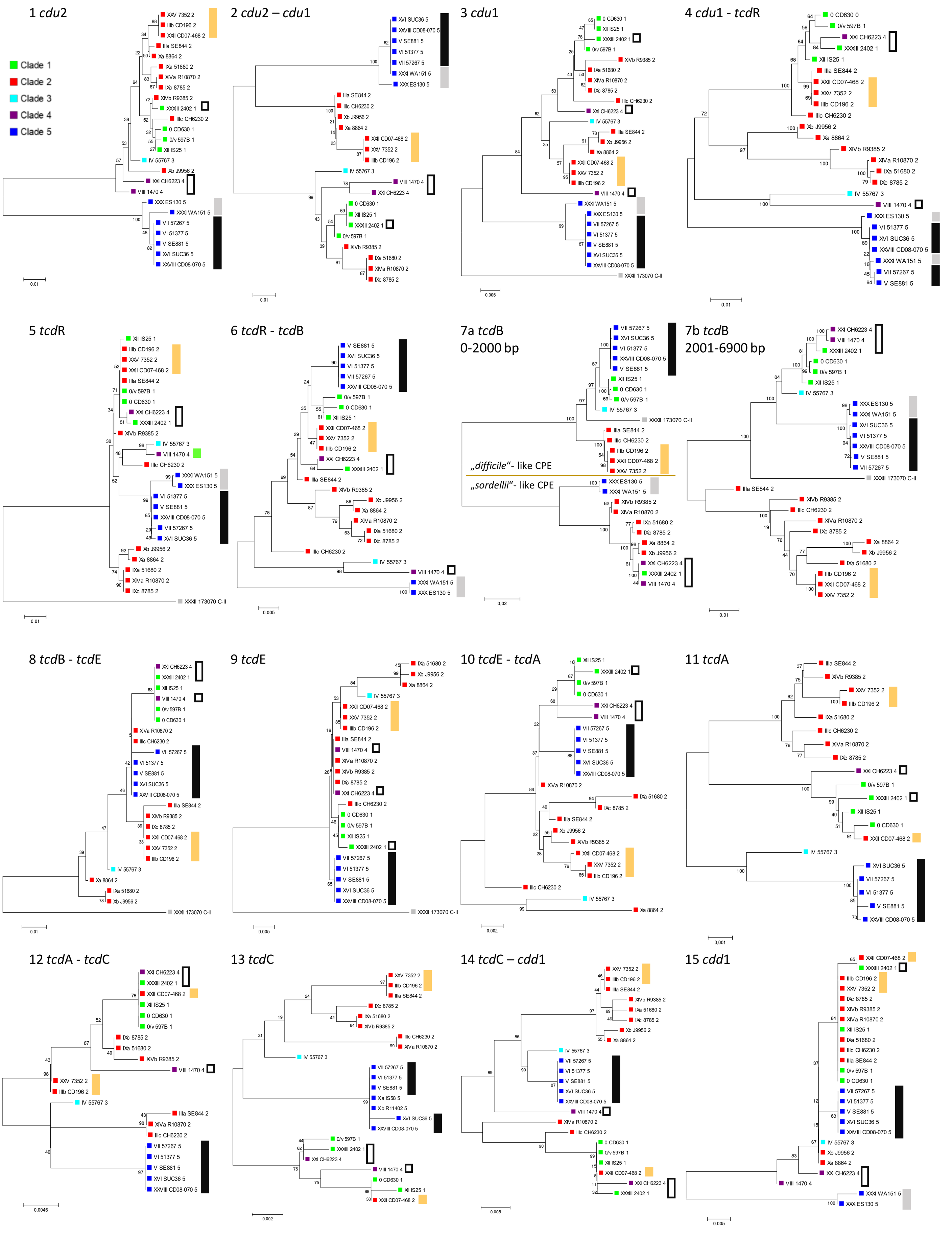

Figure S1. Neighbor-joining phylogenetic trees for the PaLoc genes and intergenic regions. Colored shapes before names indicate the clade. Colored boxes denote toxinotypes which phylogenetic relationship changes along the PaLoc region.

Table S1. Density and patterns of mutation distribution along the PaLoc. SNP density in 50 or 100 – bp windows along the PaLoc. Nucleotide sequences of the entire PaLoc were compared to a reference strain CD630 (toxinotype 0).

Middle	***cdu2***	VIII_C000	XI_C000	XXIII_C	XXX_C00	XXXI_C0	XIVa_C0001	XIVb_C0001	Xa_C000	XVII_C00	IXa_C000	IXc_C000	IIIb_CD19	XXII_C00	XXV_C00	IIIc_C000	IIIa_C000	XXXII_C0	0_CD630	0_v_C000	XII_C000	IV_C0001	V_C0001	VI_C0001	VII_C000	XVI_C000	XXVIII_C00015444
25		0	0	0	50	50	0	0	0	1	0	1	0	0	0	0	1		0	0	0	0	50	50	50	50	50
75		3	2	1	50	50	0	1	3	6	0	1	3	3	3	0	6		0	1	0	2	50	50	50	50	50
125		1	1	0	50	50	1	0	2	3	1	1	3	3	3	1	3		0	0	0	1	50	50	50	50	50
175		0	0	0	50	50	0	0	0	2	0	0	0	0	0	0	2		0	0	0	0	50	50	50	50	50
225		2	2	1	50	50	0	1	0	2	0	0	0	0	0	0	0		0	0	0	1	50	50	50	50	50
275		1	1	1	50	50	1	1	1	2	1	2	1	1	1	1	1		0	1	1	2	50	50	50	50	50
325		2	1	1	50	50	0	1	0	2	0	0	0	0	0	0	0		0	1	1	2	50	50	50	50	50
375		1	1	1	50	50	0	1	1	1	0	0	1	1	1	0	0		0	0	1	0	50	50	50	50	50
425		2	0	0	50	50	0	0	0	0	0	0	0	0	0	0	0		0	0	0	0	50	50	50	50	50
475		0	0	0	50	50	0	0	0	2	0	0	0	0	0	0	0		0	0	0	0	50	50	50	50	50
525		0	0	0	50	50	0	0	0	0	0	0	0	0	0	0	0		0	0	0	0	50	50	50	50	50
575		1	1	1	49	49	2	1	0	1	3	2	0	0	0	0	2		0	0	0	2	49	49	49	49	49
625		2	1	0	3	3	1	0	0	2	2	1	0	0	0	0	1		0	0	0	0	3	3	3	3	3
675		2	2	0	9	9	1	0	1	2	1	1	1	1	1	0	1		0	0	0	1	9	9	9	9	9
725		2	1	1	10	10	1	0	1	2	1	1	1	1	1	0	1		0	0	0	2	6	10	6	10	10
775		1	1	0	50	50	0	0	0	0	0	0	0	0	0	0	1		0	0	0	1	47	50	47	50	50
825		1	1	0	50	50	2	0	2	1	2	2	2	2	2	2	2		0	3	0	1	50	50	50	50	50
875		0	0	0	50	50	1	0	0	0	1	1	0	0	0	0	0		0	0	0	0	50	50	50	50	50
925		1	0	0	43	43	0	0	0	0	0	0	0	0	0	0	0		0	0	0	0	50	43	50	43	43
975		2	2	1	10	10	2	1	2	2	2	2	2	2	2	0	2		0	0	0	2	10	10	10	10	10
1025		1	1	1	8	9	1	1	1	1	1	1	1	1	1	1	1		0	1	1	0	8	8	8	8	8
1075		1	1	0	4	5	1	0	1	1	1	1	3	3	3	2	1		0	0	0	0	5	5	5	5	5
1125		0	0	0	7	7	1	0	0	1	0	1	0	0	0	0	0		0	1	0	0	7	7	7	7	7
1175		0	2	0	4	5	3	2	11	11	3	3	11	11	11	11	11		0	0	0	0	4	4	4	4	4

Middle	***cdu2-cdu1***	VIII_C000	XI_C000	XXIII_C	XXX_C00	XXXI_C0	XIVa_C0001	XIVb_C0001	Xa_C000	XVII_C00	IXa_C000	IXc_C000	IIIb_CD19	XXII_C00	XXV_C00	IIIc_C000	IIIa_C000	XXXII_C0	0_CD630	0_v_C000	XII_C000	IV_C0001	V_C0001	VI_C0001	VII_C000	XVI_C000	XXVIII_C00015444
25		7	4	0	14	15	4	3	7	7	4	4	7	7	7	7	7	50	0	1	0	5	15	15	15	15	15
75		1	0	0	7	6	0	0	5	5	0	0	6	6	6	5	5	50	0	0	0	0	6	6	6	6	6
125		2	2	0	5	5	1	0	5	5	1	1	6	6	6	5	5	10	0	0	0	1	5	5	5	5	5
175		0	0	0	1	1	0	0	1	1	0	0	1	1	1	1	1	4	0	0	0	1	1	1	1	1	1

Middle	***cdu1***	VIII_C000	XI_C000	XXIII_C	XXX_C00	XXXI_C0	XIVa_C0001	XIVb_C0001	Xa_C000	XVII_C00	IXa_C000	IXc_C000	IIIb_CD19	XXII_C00	XXV_C00	IIIc_C000	IIIa_C000	XXXII_C0	0_CD630	0_v_C000	XII_C000	IV_C0001	V_C0001	VI_C0001	VII_C000	XVI_C000	XXVIII_C00015444
25		2	0	0	2	2	0	0	1	1	0	0	1	1	1	1	1	1	0	0	0	2	2	2	2	2	2
75		1	1	1	2	2	1	1	1	1	1	1	1	1	1	1	1	1	0	1	0	1	2	2	2	2	2
125		3	2	0	2	2	0	0	3	3	0	0	3	3	3	1	3	4	0	0	0	2	2	2	2	2	2
175		4	1	1	3	4	0	0	1	1	0	0	1	1	1	1	1	5	0	0	0	2	3	3	3	3	3
225		2	0	0	3	1	0	1	1	1	0	0	1	1	1	0	1	5	0	0	0	1	3	3	3	3	3
275		0	0	0	1	1	0	2	2	3	0	0	0	0	0	1	3	4	0	0	0	1	1	1	1	1	1
325		0	1	0	5	5	1	1	1	1	1	1	0	0	0	0	2	3	0	0	0	1	5	5	5	5	5
375		2	0	0	4	4	1	1	1	1	1	1	1	1	1	1	1	3	0	0	0	1	4	4	4	4	4

Middle	***cdu1-tcdR***	VIII_C000	XI_C000	XXIII_C	XXX_C00	XXXI_C0	XIVa_C0001	XIVb_C0001	Xa_C000	XVII_C00	IXa_C000	IXc_C000	IIIb_CD19	XXII_C00	XXV_C00	IIIc_C000	IIIa_C000	XXXII_C0	0_CD630	0_v_C000	XII_C000	IV_C0001	V_C0001	VI_C0001	VII_C000	XVI_C000	XXVIII_C00015444
25		1	0	0	0	0	4	0	1	0	4	4	0	0	0	0	0	50	0	0	0	0	0	0	0	0	0
75		1	0	0	3	3	3	3	1	1	3	3	1	1	1	1	1	50	0	0	0	1	4	3	4	3	3
125		2	0	1	3	3	8	8	1	1	8	8	1	1	1	1	1	50	0	0	0	1	3	3	3	3	3
175		2	0	0	3	3	1	1	2	2	1	1	1	1	1	1	1	50	0	7	0	1	3	3	3	3	3
225		3	0	0	2	2	1	1	1	1	1	1	0	0	0	0	0	50	0	0	0	1	2	2	2	2	2
275		2	1	0	3	3	2	2	2	2	2	2	0	0	0	0	0	50	0	0	0	5	3	3	3	3	3
325		7	1	0	9	9	4	5	3	3	4	4	0	0	0	0	0	50	0	0	0	6	9	9	9	9	9
375		3	0	1	7	7	4	3	1	1	4	4	0	0	0	2	0	50	0	0	2	6	7	7	7	7	7
425		5	0	0	8	8	4	3	3	3	4	4	0	0	0	0	0	50	0	0	0	5	8	8	8	8	8
475		1	0	0	3	3	2	2	1	1	2	2	1	1	1	0	1	50	0	0	0	1	3	3	3	3	3
525		9	5	0	2	4	2	2	1	1	2	2	0	0	0	0	0	50	0	0	0	8	3	3	3	3	3
575		9	1	1	10	9	5	4	4	4	6	6	2	2	2	1	2	50	0	3	2	8	9	9	9	9	9
625		11	0	1	8	8	7	5	6	6	8	7	2	2	2	2	3	50	0	0	1	8	8	8	8	8	8
675		22	3	0	6	6	9	8	5	5	9	9	0	0	0	2	0	50	0	1	1	5	6	6	6	6	6
725		10	0	1	5	5	4	4	7	6	4	4	1	1	1	0	1	50	0	0	1	7	5	5	5	5	5
775		7	3	3	4	4	4	4	5	5	4	4	2	2	2	2	2	50	0	0	1	5	4	4	4	4	4
825		10	2	2	7	8	7	7	10	9	7	7	0	0	0	0	0	50	0	0	0	10	8	8	8	8	8
875		4	2	2	7	7	3	3	5	4	3	3	1	1	1	3	0	29	0	1	0	4	7	7	7	7	7
925		0	0	0	0	0	0	0	0	0	0	0	0	0	0	0	0	0	0	0	0	0	0	0	0	0	0

Middle	***tcdR***	VIII_C000	XI_C000	XXIII_C	XXX_C00	XXXI_C0	XIVa_C0001	XIVb_C0001	Xa_C000	XVII_C00	IXa_C000	IXc_C000	IIIb_CD19	XXII_C00	XXV_C00	IIIc_C000	IIIa_C000	XXXII_C0	0_CD630	0_v_C000	XII_C000	IV_C0001	V_C0001	VI_C0001	VII_C000	XVI_C000	XXVIII_C00015444
25		3	0	0	1	1	1	1	1	1	1	1	0	0	0	1	0	5	0	0	0	1	1	1	1	1	1
75		1	0	0	0	0	0	0	2	2	0	0	0	0	0	1	0	3	0	0	0	1	0	0	0	0	0
125		0	0	0	2	2	0	0	0	0	0	0	0	0	0	1	0	5	0	0	1	0	2	2	2	2	2
175		1	0	0	0	0	0	0	0	1	0	0	0	0	0	1	0	11	0	0	0	1	0	0	1	0	0
225		2	0	0	3	3	1	1	1	1	1	1	0	0	0	2	0	7	0	0	0	2	3	3	3	3	3
275		0	0	0	4	4	0	0	0	0	0	0	0	0	0	0	0	5	0	0	0	0	4	4	4	4	4
325		3	1	2	2	1	3	0	4	4	3	3	0	0	0	1	0	10	0	0	0	3	1	1	1	1	1
375		1	1	1	2	2	0	0	0	0	0	0	0	0	0	2	0	10	0	0	0	2	0	0	0	0	0
425		1	0	0	0	0	0	0	0	0	0	0	0	0	0	0	0	3	0	0	0	1	0	0	0	0	0
475		0	0	0	3	3	1	0	0	0	1	1	0	0	0	0	0	3	0	0	0	1	0	0	0	1	0
525		0	0	0	0	0	0	0	0	0	0	0	0	0	0	0	0	4	0	0	0	0	0	0	0	0	0
575		0	0	0	1	1	0	0	0	0	0	0	0	0	0	0	0	1	0	0	0	0	0	0	0	0	0

Middle	***tcdR-tcdB***	VIII_C000	XI_C000	XXIII_C	XXX_C00	XXXI_C0	XIVa_C0001	XIVb_C0001	Xa_C000	XVII_C00	IXa_C000	IXc_C000	IIIb_CD19	XXII_C00	XXV_C00	IIIc_C000	IIIa_C000	XXXII_C0	0_CD630	0v_C0001	XII_C000	IV_C0001	V_C0001	VI_C0001	VII_C000	XVI_C000	XXVIII_C00015444
15		4	1	1	7	7	2	2	2	2	2	2	2	2	2	2	2	25	0	0	0	3	1	1	1	1	1
45		1	0	1	1	1	1	0	1	1	1	1	0	0	0	0	0	30	0	0	0	1	0	0	0	0	0
75		2	1	1	1	1	0	0	0	0	0	0	0	0	0	0	0	30	0	1	0	2	0	0	0	0	0
105		4	1	1	3	3	1	1	2	2	2	2	0	0	0	1	1	30	0	0	0	4	0	0	0	0	0
135		2	0	0	2	2	2	2	1	1	2	2	0	0	0	3	0	30	0	0	0	3	0	0	0	0	0
165		2	0	0	1	1	2	2	2	2	2	2	0	0	0	1	0	30	0	0	0	1	1	1	1	1	1
195		2	1	1	1	1	1	1	1	1	1	1	1	1	1	1	1	30	0	1	1	1	2	2	2	2	2
225		0	0	0	2	2	0	0	0	0	0	0	0	0	0	0	0	30	0	0	0	0	0	0	0	0	0
255		1	0	0	2	2	0	0	0	1	0	0	0	0	0	0	0	30	0	0	0	0	0	0	0	0	0
285		0	0	0	0	0	0	0	0	0	0	0	0	0	0	0	0	18	0	0	0	0	0	0	0	0	0
315		0	0	1	0	0	0	0	0	0	0	0	0	0	0	0	0	1	0	0	0	0	0	0	0	0	0
			A		A+B	A+B	A+C*	A+D	A+C*	A+C*	A+C*	A+?		C		D	E	F							B		

Middle	***tcdB***	VIII_C000	XI_C000	XXIII_C	XXX_C00	XXXI_C0	XIVa_C0001	XIVb_C0001	Xa_C000	Xb_C000	IXa_C000	IXc_C000	IIIb_CD19	XXII_C00	XXV_C00	IIIc_C000	IIIa_C000	XXXII_C0	0_CD630	0_v_C000	XII_C000	IV_C0001	V_C0001	VI_C0001	VII_C000	XVI_C000	XXVIII_C00015444
50	**GT**	6	6	6	1	1	6	6	6	6	6	6	4	4	4	1	1	8	0	0	0	0	1	1	1	1	1
150	**GT**	6	6	7	2	2	7	7	7	7	7	7	1	1	1	1	1	6	0	0	0	0	0	0	0	0	0
250	**GT**	14	14	14	0	0	14	14	13	13	14	14	1	1	1	1	1	12	0	1	0	0	0	0	0	0	0
350	**GT**	5	5	5	1	1	5	5	5	5	5	5	0	0	0	0	0	6	0	0	0	1	0	0	0	0	0
450	**GT**	10	10	10	9	9	10	10	10	10	10	10	2	2	2	2	2	7	0	0	0	3	2	2	2	3	2
550	**GT**	12	12	12	11	11	13	13	12	12	12	13	8	8	8	8	8	8	0	0	0	2	2	2	2	2	2
650	**GT**	10	10	10	13	13	10	10	10	10	10	10	8	8	8	8	8	3	0	0	0	1	3	3	3	3	3
750	**GT**	11	11	11	12	12	11	11	11	11	11	11	8	8	8	8	8	2	0	0	0	1	2	2	2	2	2
850	**GT**	20	20	20	19	19	20	20	22	21	20	20	5	5	5	5	5	1	0	0	0	1	0	0	0	0	0
950	**GT**	27	27	27	24	25	26	26	26	26	26	26	6	6	6	6	6	2	0	0	1	2	2	2	2	2	2
1050	**GT**	14	14	14	13	13	13	14	13	13	13	13	6	6	6	6	6	4	0	1	0	2	2	2	2	2	2
1150	**GT**	24	24	24	20	20	23	23	24	24	23	23	2	2	2	2	2	0	0	0	0	0	0	0	0	0	0
1250	**GT**	22	22	22	23	23	22	22	22	22	22	22	6	6	6	6	6	3	0	0	1	3	2	2	2	2	2
1350	**GT**	33	33	33	36	36	33	33	33	33	33	33	0	0	0	0	0	0	0	0	0	0	1	1	2	1	1
1450	**GT**	31	31	31	33	33	31	31	31	31	31	31	2	2	2	2	2	3	0	0	0	1	0	0	0	0	0
1550	**GT**	24	24	24	23	23	22	22	24	24	23	22	0	0	0	0	0	0	0	0	0	1	2	2	2	2	2
1650	**GT**	13	13	13	13	12	12	12	13	13	11	12	8	8	8	8	7	7	0	0	0	0	0	0	0	0	0
1750	**AP**	12	12	12	10	10	11	11	12	12	11	11	0	0	0	0	0	4	0	0	0	2	1	1	1	1	1
1850	**AP**	14	14	14	9	9	0	0	14	14	14	14	0	0	0	0	0	3	0	1	0	1	3	3	3	3	3
1950	**AP**	10	10	10	7	7	1	1	12	12	12	12	1	1	1	1	1	4	0	0	0	1	2	2	2	2	2
2050	**AP**	11	11	11	4	4	4	4	4	4	4	4	4	4	4	4	4	4	4	4	4	4	4	4	4	4	4
2150	**AP**	9	9	8	2	2	8	0	8	8	8	7	7	7	7	7	0	4	0	0	0	0	2	2	2	2	2
2250	**AP**	3	3	3	0	0	0	0	4	4	1	0	0	0	0	0	0	1	0	0	0	0	1	1	1	1	1
2350	**AP**	4	4	0	1	2	3	3	3	3	3	3	3	3	3	3	3	3	0	2	1	1	1	1	1	1	1
2450	**TR**	1	1	0	3	3	2	2	2	2	2	2	2	2	2	2	2	2	0	0	0	0	3	3	3	3	3
2550	**TR**	12	12	0	2	2	10	10	11	11	11	10	11	11	11	10	10	2	0	0	0	3	2	2	2	2	2
2650	**TR**	1	0	0	14	14	8	8	9	9	9	8	9	9	9	9	8	1	0	0	1	7	14	14	14	14	14
2750	**TR**	0	0	0	11	11	5	3	5	5	3	5	3	3	3	3	5	2	0	0	0	1	11	11	11	11	11
2850	**TR**	0	0	0	5	5	6	3	7	6	3	6	3	3	3	3	5	2	0	0	0	2	5	5	5	5	5
2950	**TR**	1	1	0	5	5	3	2	4	4	3	3	2	2	2	2	3	2	0	1	1	2	5	5	5	5	5
3050	**TR**	0	0	0	1	1	3	1	6	6	3	2	2	2	2	1	2	1	0	0	0	1	1	1	1	1	1
3150	**TR**	0	0	0	3	3	2	1	8	9	2	1	2	2	2	0	0	2	0	0	0	1	3	3	3	3	3
3250	**TR**	0	0	0	4	4	3	0	7	7	4	3	3	3	3	0	0	6	0	0	0	1	4	4	4	4	4
3350	**TR**	0	0	0	3	3	15	0	18	18	15	15	15	15	15	0	0	8	0	0	0	0	3	3	3	3	3
3450	**TR**	1	1	0	3	3	9	0	8	8	9	9	9	9	9	0	0	8	0	0	0	0	3	3	3	3	3
3550	**TR**	0	0	0	10	10	5	1	8	8	4	5	1	1	1	1	1	7	0	0	0	3	10	10	10	10	10
3650	**TR**	0	0	1	2	2	8	0	13	13	7	8	0	0	0	0	0	2	0	0	0	1	2	2	2	2	2
3750	**TR**	0	0	0	6	6	6	0	9	9	8	6	0	0	0	0	0	4	0	0	0	3	6	6	6	6	6
3850	**TR**	0	0	0	6	6	5	1	6	6	5	2	1	1	1	1	0	2	0	0	0	0	6	5	5	5	5
3950	**TR**	0	0	0	5	5	9	9	6	6	9	5	9	9	9	9	0	2	0	0	0	2	5	5	5	5	5
4050	**TR**	0	0	0	1	1	13	13	13	13	15	13	13	13	13	13	0	0	0	0	0	0	1	1	1	1	1
4150	**TR**	0	0	0	9	8	18	18	17	17	18	18	18	18	18	18	1	8	0	0	1	2	8	8	8	8	8
4250	**TR**	0	0	0	1	1	8	8	8	8	8	8	8	8	8	8	0	3	0	0	0	1	1	1	1	1	1
4350	**TR**	0	0	0	0	0	13	13	12	12	13	13	13	13	13	13	1	8	0	0	0	2	0	0	0	0	0
4450	**TR**	0	0	0	0	0	9	11	11	11	11	8	9	9	9	9	0	6	0	0	0	2	0	0	0	0	0
4550	**TR**	0	1	0	0	0	15	12	12	12	12	0	15	15	15	15	1	10	0	0	0	1	0	0	0	0	0
4650	**TR**	0	0	0	3	3	12	13	13	13	13	0	12	12	12	12	0	12	0	0	0	2	3	3	3	3	3
4750	**TR**	1	1	1	4	4	7	10	10	10	9	1	7	7	7	7	1	19	0	1	0	2	4	4	4	4	4
4850	**TR**	1	0	0	3	3	9	8	8	8	8	4	7	7	7	9	0	15	0	0	0	1	3	3	3	3	3
4950	**TR**	0	0	0	2	2	4	4	5	5	5	4	4	4	4	4	0	6	0	0	0	1	2	2	2	2	2
5050	**TR**	0	0	0	0	0	7	9	9	9	9	9	9	9	9	7	0	9	0	0	0	1	0	0	0	0	0
5150	**TR**	1	1	0	2	2	8	9	9	9	9	9	9	9	9	8	7	4	0	0	0	1	2	2	2	2	2
5250	**TR**	0	0	0	0	0	9	8	8	7	8	8	8	8	8	9	8	10	0	0	0	0	0	0	0	0	0
5350	**TR**	0	0	0	5	5	20	20	21	20	20	20	21	21	21	20	20	10	0	0	1	4	6	6	6	6	6
5450	**TR**	0	0	0	1	1	12	12	14	13	13	12	12	12	12	12	12	12	0	0	0	4	1	1	1	1	1
5550	**TR**	0	0	0	5	5	11	11	10	11	11	11	11	11	11	11	11	6	0	0	0	2	5	5	5	5	5
5650	**RB**	0	1	0	4	4	11	10	10	11	12	11	11	11	11	11	11	3	0	0	0	1	5	5	5	5	5
5750	**RB**	0	1	1	1	1	10	10	8	10	9	10	10	10	10	10	10	1	0	0	1	0	1	1	1	0	1
5850	**RB**	1	1	0	3	3	14	15	15	15	15	15	15	15	15	15	15	2	0	0	1	2	3	3	3	3	3
5950	**RB**	1	1	1	7	6	11	11	12	11	11	11	11	11	11	11	11	4	0	1	1	2	6	6	6	6	6
6050	**RB**	0	0	0	6	6	12	12	12	12	11	12	12	12	12	12	12	3	0	0	1	1	6	6	7	6	6
6150	**RB**	0	0	0	5	5	10	10	10	11	10	10	10	10	10	10	10	6	0	0	0	0	5	5	5	5	5
6250	**RB**	0	0	0	3	3	6	6	8	6	7	6	6	6	6	6	8	7	0	0	1	1	2	2	2	2	2
6350	**RB**	0	0	0	5	5	3	15	15	15	16	15	15	15	15	4	15	9	0	0	0	2	4	4	4	4	4
6450	**RB**	0	0	0	6	6	5	11	13	10	11	11	11	11	11	5	13	5	0	0	4	4	6	6	6	6	6
6550	**RB**	0	0	0	4	5	2	4	6	6	4	4	4	4	4	2	6	9	0	0	1	1	5	5	5	5	5
6650	**RB**	1	0	0	1	1	2	4	11	11	4	4	4	4	4	2	11	6	0	0	0	0	2	1	1	1	1
6750	**RB**	0	0	1	2	2	11	10	9	9	9	10	10	10	10	11	10	9	0	0	0	2	2	2	2	2	2
6850	**RB**	0	0	0	4	4	8	11	8	8	11	12	12	12	12	8	12	9	0	0	0	0	4	4	4	4	4
6950	**RB**	0	0	0	5	5	2	2	2	2	1	1	2	2	2	2	2	8	0	0	0	0	1	0	0	0	0
7050	**RB**	0	0	0	10	10	0	0	0	1	4	1	0	0	0	0	1	6	0	0	0	0	0	0	0	0	0
7150	**RB**	0	0	0	0	0	0	0	0	0	0	0	0	0	0	0	0	2	0	0	0	0	0	0	0	0	0

Middle	***tcdB-tcdE***	VIII_C000	XXI_C000	XXXIII_C0	XXX_C00	XXXI_C0	XIVa_C0001	XIVb_C0001	Xa_C000	XVII_C00	IXa_C000	IXc_C000	IIIb_CD19	XXII_C00	XXV_C00	IIIc_C000	IIIa_C000	XXXII_C0	0_CD630	0_v_C000	XII_C000	IV_C0001	V_C0001	VI_C0001	VII_C000	XVI_C000	XXVIII_C00015444
15		0	0	0			0	1	0	2	3	1	1	1	1	0	2	11	0	0	0	0	0	0	0	0	0
45		0	0	0			0	0	0	0	0	0	0	0	0	0	0	3	0	0	0	0	0	0	0	0	0
75		0	0	0			0	0	0	0	0	0	0	0	0	0	0	3	0	0	0	0	0	0	1	0	0
105		0	0	0			1	1	3	3	3	1	1	1	1	1	1	4	0	0	0	2	1	1	1	1	1
135		0	0	0			0	0	0	0	0	0	0	0	0	0	0	0	0	0	0	0	0	0	0	0	0

Middle	***tcdE***	VIII_C000	XXI_C000	XXXIII_C0	XXX_C00	XXXI_C0	XIVa_C0001	XIVb_C0001	Xa_C000	XVII_C00	IXa_C000	IXc_C000	IIIb_CD19	XXII_C00	XXV_C00	IIIc_C000	IIIa_C000	XXXII_C0	0_CD630	0_v_C000	XII_C000	IV_C0001	V_C0001	VI_C0001	VII_C000	XVI_C000	XXVIII_C00015444
25		0	0	0			0	0	3	0	3	0	0	0	0	0	0	5	0	0	0	0	1	1	1	1	1
75		0	0	0			0	0	1	1	1	0	0	0	0	1	0	2	0	0	0	0	0	0	0	0	0
125		0	0	0			0	0	0	1	0	0	0	0	0	0	0	2	0	0	0	0	0	0	0	0	0
175		0	0	0			0	0	2	2	2	0	0	0	0	0	0	4	0	0	0	0	0	0	0	0	0
225		0	0	0			0	0	0	0	0	0	0	0	0	0	0	1	0	0	0	1	0	0	0	0	0
275		0	0	0			0	0	1	1	2	0	0	0	0	0	0	2	0	0	0	0	0	0	0	0	0
325		0	0	0			0	0	1	1	1	0	0	0	0	1	0	6	0	0	0	1	0	0	0	0	0
375		0	0	0			0	0	1	1	1	0	1	1	1	0	0	3	0	0	0	1	0	0	0	0	0
425		0	0	0			0	0	1	1	1	0	0	0	0	0	0	6	0	0	0	1	0	0	0	0	0
475		1	1	1			1	1	2	2	2	1	1	1	1	0	1	9	0	0	0	1	1	1	1	1	1
525		0	0	0			0	0	0	0	0	0	0	0	0	0	0	1	0	0	0	0	0	0	0	0	0

Middle	***tcdE-tcdA***	VIII_C000	XXI_C000	XXXIII_C0	XXX_C00	XXXI_C0	XIVa_C0001	XIVb_C0001	Xa_C000	XVII_C00	IXa_C000	IXc_C000	IIIb_CD19	XXII_C00	XXV_C00	IIIc_C000	IIIa_C000	XXXII_C0	0_CD630	0-v_C000	XII_C000	IV_C0001	V_C0001	VI_C0001	VII_C000	XVI_C000	XXVIII_C00015444
25		0	1	0			0	0	1	0	0	0	1	1	1	0	0	7	0	0	0	0	1	1	1	1	1
75		0	0	0			1	1	4	1	2	1	1	1	1	5	1	13	0	0	0	4	1	1	1	1	1
125		0	0	0			0	0	3	0	2	2	0	0	0	1	1	10	0	0	0	3	0	0	0	0	0
175		1	1	0			1	1	1	1	1	1	1	1	1	1	1	11	0	0	0	1	1	1	1	1	1
225		0	0	0			0	0	4	0	1	1	0	0	0	0	0	16	0	0	0	3	0	0	0	0	0
275		0	0	0			0	1	0	0	0	0	0	0	0	0	0	8	0	0	0	1	0	0	0	0	0
325		0	0	0			0	0	20	0	0	0	0	0	0	0	0	32	0	0	0	18	0	0	0	0	0
375		1	1	0			1	1	50	1	1	1	1	1	1	1	1	50	0	0	0	4	1	1	1	1	1
425		0	0	0			0	0	20	0	0	0	0	0	0	0	0	50	0	0	0	0	0	0	0	0	0
475		10	1	0			0	0	1	0	1	0	0	0	0	0	0	50	0	0	0	0	0	0	0	0	0
525		0	0	0			0	0	1	0	0	0	0	0	0	0	0	50	0	0	0	1	0	0	0	0	0
575		1	0	0			0	0	0	0	0	0	1	1	1	0	0	50	0	0	0	0	0	0	0	0	0
625		0	0	1			0	0	0	0	0	0	0	0	0	0	0	50	0	0	0	0	1	1	1	1	1
675		0	0	0			0	0	0	0	0	0	0	0	0	0	0	50	0	0	0	0	0	0	0	0	0
725		0	0	0			0	1	0	1	1	1	1	0	1	0	0	27	0	0	0	0	0	0	0	0	0

Middle	***tcdA***	VIII_C000	XXI_C000	XXXIII_C0	XXX_C00	XXXI_C0	XIVa_C0001	XIVb_C0001	Xa_C000	XVII_C00	IXa_C000	IXc_C000	IIIb_CD19	XXII_C00	XXV_C00	IIIc_C000	IIIa_C000	XXXII_C0	0_CD630	0_v_C000	XII_C000	IV_C0001	V_C0001	VI_C0001	VII_C000	XVI_C000	XXVIII_C00015444	XIa_C000	XIb_C00015435
50	**GT**	0	0	0			0	0	1	0	0	1	0	0	0	0	0		0	1	0	0	0	0	0	0	0	100	100
150	**GT**	2	1	0			1	1	1	1	1	1	1	0	1	1	1		0	0	0	1	1	1	1	1	1	100	100
250	**GT**	1	0	0			1	1	1	1	1	1	1	0	1	0	2		0	0	0	1	0	0	0	0	0	100	100
350	**GT**	1	1	1			0	1	1	1	0	0	0	0	0	0	1		0	1	0	0	0	0	0	0	0	100	100
450	**GT**	2	1	0			1	1	3	1	1	1	1	0	1	1	1		0	0	0	4	1	1	1	1	1	100	100
550	**GT**	1	0	0			0	0	1	1	1	0	0	0	0	0	0		0	0	0	0	1	1	1	1	1	100	100
650	**GT**	1	1	0			1	1	2	2	1	1	1	0	1	1	1		0	0	0	1	1	1	1	1	1	100	100
750	**GT**	2	1	0			0	1	5	5	0	1	1	0	1	1	0		0	0	0	3	2	2	2	2	2	100	100
850	**GT**	1	1	0			0	0	0	0	0	0	0	0	0	0	0		0	0	0	1	0	0	0	0	0	100	100
950	**GT**	0	1	0			0	1	0	0	0	1	1	0	1	0	0		0	0	0	0	0	0	0	0	0	100	100
1050	**GT**	0	0	0			1	0	0	0	0	0	0	0	0	0	0		0	0	0	0	0	0	0	0	0	100	100
1150	**GT**	1	0	0			0	0	1	2	0	0	0	0	0	0	0		0	0	0	0	0	0	0	0	0	100	100
1250	**GT**	1	2	0			3	3	3	3	3	0	3	0	3	0	3		0	0	0	0	1	1	1	2	1	100	100
1350	**GT**	1	0	1			0	0	2	2	0	0	0	0	0	0	0		0	0	0	0	0	0	0	0	0	100	100
1450	**GT**	1	1	1			1	1	1	1	1	1	1	0	1	1	1		0	1	0	1	1	1	1	1	1	100	100
1550	**GT**	1	0	0			0	0	0	0	0	0	0	0	0	0	0		0	0	0	0	0	0	0	0	0	100	100
1650	**AP**	1	2	0			0	0	4	4	0	0	0	0	0	0	0		0	0	0	2	2	2	2	2	2	100	100
1750	**AP**	0	0	0			0	2	2	2	0	0	0	0	0	0	0		0	0	0	2	3	3	3	3	3	100	100
1850	**AP**	2	0	0			1	1	1	1	1	1	1	0	1	1	1		0	0	0	2	2	2	2	2	2	100	100
1950	**AP**	0	0	0			0	0	1	1	0	0	0	0	0	0	0		0	0	0	0	1	1	1	2	1	100	100
2050	**AP**	0	0	0			1	1	2	2	1	1	1	0	1	1	1		0	0	0	1	1	1	1	1	1	100	100
2150	**AP**	2	1	0			3	2	3	3	2	3	2	0	2	3	2		0	1	1	2	2	2	2	2	2	100	100
2250	**AP**	1	0	0			2	2	2	2	2	2	2	0	2	2	2		0	0	0	2	3	3	3	3	3	100	100
2350	**TR**	0	0	0			5	4	2	2	4	5	4	0	4	5	4		0	0	0	2	3	3	3	3	3	100	100
2450	**TR**	0	0	0			0	0	1	1	0	0	0	0	0	0	0		0	0	0	0	3	3	3	3	3	57	57
2550	**TR**	0	1	0			2	2	1	1	2	2	2	0	2	2	2		0	0	0	3	2	2	2	2	2	2	2
2650	**TR**	1	0	0			1	1	0	0	1	1	1	0	1	1	1		0	0	0	1	1	1	1	1	1	1	1
2750	**TR**	1	0	0			2	3	2	2	2	2	2	0	2	2	2		0	0	0	2	2	2	2	2	2	2	2
2850	**TR**	1	0	0			1	0	61	61	0	1	0	0	0	1	0		0	0	0	2	3	3	3	3	3	3	3
2950	**TR**	0	0	0			0	0	100	100	0	0	0	0	0	0	0		0	0	0	0	1	1	1	1	1	1	1
3050	**TR**	0	0	0			0	0	100	100	0	0	0	0	0	0	0		0	0	0	1	1	1	1	1	1	1	1
3150	**TR**	1	1	0			1	1	100	100	1	1	1	0	1	1	1		0	0	1	1	1	1	1	1	1	1	1
3250	**TR**	1	0	0			1	1	100	100	1	1	1	0	1	1	1		0	0	0	1	1	1	1	1	1	1	1
3350	**TR**	1	0	0			0	0	100	100	0	0	2	0	2	0	0		0	0	0	2	2	2	2	2	2	2	2
3450	**TR**	0	1	0			0	0	100	100	0	0	0	0	0	0	0		0	0	0	1	1	1	1	1	1	1	1
3550	**TR**	0	0	1			3	3	100	100	2	3	2	0	2	3	2		0	0	1	0	0	0	0	0	0	0	0
3650	**TR**	0	0	0			4	4	100	100	4	4	3	0	3	4	4		0	0	0	2	2	2	2	2	2	2	2
3750	**TR**	3	2	2			5	4	100	100	4	5	6	0	6	4	5		0	2	2	6	6	6	6	6	6	6	6
3850	**TR**	0	0	0			3	4	100	100	4	3	4	0	4	3	4		0	1	0	2	2	2	2	2	2	2	2
3950	**TR**	0	0	0			1	2	100	100	2	1	1	0	1	2	2		0	0	0	0	1	1	1	1	1	1	1
4050	**TR**	1	0	0			1	1	100	100	1	1	1	0	1	1	1		0	0	0	0	1	1	1	1	1	1	1
4150	**TR**	1	0	0			4	4	100	100	4	4	4	0	4	4	4		0	0	0	2	1	1	1	1	1	1	1
4250	**TR**	2	0	0			0	0	100	100	0	0	0	0	0	0	0		0	0	0	2	2	2	2	2	2	2	2
4350	**TR**	3	1	1			1	1	100	100	1	1	2	1	2	1	1		0	2	1	2	2	2	2	2	2	2	2
4450	**TR**	1	0	0			1	1	100	100	1	1	1	0	1	2	1		0	0	0	1	1	1	1	1	1	1	1
4550	**TR**	0	0	0			0	0	100	100	0	0	1	0	1	0	0		0	0	0	0	0	0	0	0	0	0	0
4650	**TR**	2	0	1			0	0	100	100	0	0	0	0	0	0	0		0	0	0	0	2	2	2	2	2	2	2
4750	**TR**	1	1	0			1	1	100	100	1	1	1	0	1	1	1		0	0	0	0	1	1	1	1	1	1	1
4850	**TR**	0	0	0			0	0	100	100	0	0	0	0	0	0	0		0	0	0	1	1	1	1	1	1	1	1
4950	**TR**	1	0	0			0	0	100	100	1	0	0	0	0	0	0		0	0	0	1	0	0	0	0	0	0	0
5050	**TR**	1	0	0			0	0	100	100	0	0	0	0	0	0	0		0	0	0	1	1	1	1	1	1	1	1
5150	**TR**	1	0	1			0	0	100	100	0	0	0	0	0	0	0		0	0	0	0	0	0	0	0	0	0	0
5250	**TR**	0	0	0			0	0	100	100	0	0	0	0	0	0	0		0	0	0	0	0	0	0	0	0	0	0
5350	**TR**	2	0	1			0	0	100	100	0	0	0	0	0	0	0		0	0	0	1	2	1	1	1	1	1	1
5450	**RB**	1	0	0			0	0	100	100	0	0	0	0	0	0	0		0	0	0	1	1	1	1	1	1	1	1
5550	**RB**	1	0	0			2	1	100	100	1	2	1	0	1	1	1		0	0	0	2	2	2	2	2	2	2	2
5650	**RB**	4	1	2			3	4	100	100	4	3	4	2	4	4	4		0	1	1	4	3	3	3	3	3	3	3
5750	**RB**	2	2	1			2	2	100	100	2	2	2	0	2	2	2		0	1	0	2	2	2	2	2	3	2	2
5850	**RB**	72	0	1			0	1	100	100	1	0	1	0	1	2	1		0	0	0	1	0	0	0	0	0	0	0
5950	**RB**	100	0	0			0	0	100	100	0	0	0	0	0	0	0		0	0	0	0	1	0	0	0	0	0	0
6050	**RB**	100	2	0			2	2	100	100	2	2	2	0	2	2	2		0	0	0	2	2	2	2	2	2	2	2
6150	**RB**	100	0	0			1	1	100	100	1	1	1	0	1	1	1		0	0	0	0	1	0	0	0	0	0	0
6250	**RB**	93	0	0			0	0	100	100	0	0	0	0	0	0	0		0	1	0	0	1	1	1	67	1	1	1
6350	**RB**	3	0	0			1	1	100	100	1	3	1	0	1	1	1		0	91	0	0	1	49	1	100	1	1	1
6450	**RB**	3	0	0			2	1	100	100	1	3	3	0	3	1	1		0	100	0	1	1	100	1	100	1	91	0
6550	**RB**	100	0	0			7	6	100	100	1	7	7	0	7	7	6		0	15	0	7	5	100	60	100	4	100	13
6650	**RB**	100	0	0			12	12	100	100	12	12	12	0	12	12	12		0	0	0	12	3	100	100	100	3	100	100
6750	**RB**	100	0	0			2	1	100	100	1	3	1	0	1	1	1		0	0	0	1	3	100	100	100	3	100	100
6850	**RB**	100	0	0			1	1	100	100	1	2	1	0	1	1	2		0	0	0	2	1	100	100	100	1	100	100
6950	**RB**	100	0	0			3	3	100	100	3	3	3	0	3	3	3		0	0	0	2	1	100	100	100	1	100	100
7050	**RB**	100	0	0			4	4	100	100	4	5	4	0	4	4	4		0	21	0	3	54	35	100	100	5	90	100
7150	**RB**	100	0	0			7	7	100	100	6	7	7	0	68	7	7		0	56	0	2	100	4	100	7	47	5	100
7250	**RB**	100	0	0			6	1	100	100	1	1	1	0	100	1	1		0	0	0	1	35	6	87	6	100	6	27
7350	**RB**	100	0	0			0	0	100	100	0	0	0	0	100	0	0		0	0	0	0	1	1	1	1	100	1	1
7450	**RB**	100	0	0			0	1	100	100	1	0	1	0	73	0	1		0	0	0	0	5	5	6	5	92	5	5
7550	**RB**	100	0	0			0	0	100	100	0	0	0	0	2	0	0		0	0	0	1	2	2	2	2	2	2	2
7650	**RB**	100	1	0			4	4	100	100	4	4	4	0	4	4	4		0	1	0	3	3	3	3	3	3	3	3
7750	**RB**	100	1	1			3	3	100	100	3	3	3	1	3	3	3		0	1	1	5	4	4	4	4	4	4	4
7850	**RB**	55	0	0			3	2	100	100	3	3	2	1	2	3	2		0	0	0	3	3	3	3	3	3	3	3
7950	**RB**	5	0	0			2	2	100	100	3	3	2	0	2	2	2		0	0	0	3	3	3	3	2	3	3	3
8050	**RB**	1	0	0			1	0	100	100	0	0	1	1	1	1	1		0	0	0	0	3	3	3	3	3	3	3
8150	**RB**	0	1	0			1	0	33	33	0	0	0	0	0	0	0		0	0	0	0	2	2	2	2	2	2	2

tcdA-tcdC

Middle	VIII_C000	XXI_C000	XXXIII_C	XXX_C00	XXXI_C0	XIVa_C0001	XIVb_C00015440			IXa_C000	IXc_C000	IIIb_CD19	XXII_C00	XXV_C00	IIIc_C000	IIIa_C000	XXXII_C0	0_CD630	0_v_C000	XII_C000	IV_C0001	V_C0001	VI_C0001	VII_C000	XVI_C000	XXVIII_C00015444	XIa_C000	XIb_C00015435
25	2	0	0			3	2			1	1	3	0	3	3	3		0	0	0	3	5	5	5	5	5	5	5
75	0	0	0			1	0			0	0	0	0	0	1	1		0	0	0	1	1	1	1	1	1	1	1
125	0	0	0			0	0			0	0	0	0	0	0	0		0	0	0	0	0	0	0	0	0	0	0
175	1	0	0			3	0			0	0	0	0	0	3	4		0	0	0	3	3	3	3	3	3	3	3
225	2	0	0			4	1			1	1	1	0	1	4	4		0	0	0	4	5	5	5	5	5	5	5
275	0	0	0			3	0			0	0	0	0	0	3	3		0	0	0	0	0	0	0	0	0	0	0
325	0	0	0			1	0			0	0	0	0	0	1	1		0	0	0	1	1	1	1	1	1	1	1

tcdC

Middle	VIII_C000	XXI_C000	XXXIII_C	XXX_C00	XXXI_C0	XIVa_C0001	XIVb_C0001	Xa_C000	XVII_C00	IXa_C000	IXc_C000	IIIb_CD19	XXII_C00	XXV_C00	IIIc_C000	IIIa_C000	XXXII_C0	0_CD630	0_v_C000	XII_C000	IV_C0001	V_C0001	VI_C0001	VII_C000	XVI_C000	XXVIII_C00015444	XIa_C000	XIb_C00015435
25	0	1	1			3	1	50	50	1	1	2	0	2	3	2		0	2	0	3	2	2	2	2	2	2	2
75	0	0	0			0	0	50	50	0	0	0	0	0	0	0		0	0	0	0	0	0	0	0	0	0	0
125	0	0	0			1	1	50	50	1	1	1	0	1	1	1		0	0	0	1	2	2	2	2	2	2	2
175	0	0	1			2	1	50	50	1	1	1	0	1	2	1		0	0	0	0	1	1	1	1	1	1	1
225	0	0	0			0	0	29	29	0	0	0	0	0	0	0		0	0	0	0	0	0	0	1	0	0	0
275	0	0	0			1	0	1	1	0	0	0	0	0	1	0		0	0	1	0	1	1	1	1	1	1	1
325	2	1	2			2	2	4	4	2	2	2	0	2	2	2		0	1	0	30	29	29	29	29	29	29	29
375	0	0	0			48	18	2	2	0	0	18	0	18	48	18		0	18	0	37	20	20	20	20	20	20	20
425	0	0	0			1	0	0	0	0	0	0	0	0	1	0		0	0	0	0	0	0	0	0	0	0	0
475	0	0	0			0	0	1	1	0	0	0	0	0	0	0		0	0	0	0	0	0	0	0	0	0	0
525	2	1	1			2	1	1	1	1	1	1	0	1	2	1		0	1	0	2	2	2	2	2	2	2	2
575	1	1	1			2	1	2	2	1	2	2	0	2	2	2		0	1	0	2	2	2	2	2	2	2	2
625	0	0	0			1	0	2	2	0	0	1	0	1	1	1		0	0	0	1	1	1	1	1	1	1	1
675	1	1	1			1	1	2	2	1	2	2	0	2	1	2		0	1	0	1	1	1	1	1	1	1	1

tcdC-cdd1

Middle	VIII_C000	XXI_C000	XXXIII_C	XXX_C00	XXXI_C0	XIVa_C0001	XIVb_C0001	Xa_C000	XVII_C00	IXa_C000	IXc_C000	IIIb_CD19	XXII_C00	XXV_C00	IIIc_C000	IIIa_C000	XXXII_C0	0_CD630	0_v_C000	XII_C000	IV_C0001	V_C0001	VI_C0001	VII_C000	XVI_C000	XXVIII_C00015444	XIa_C000	XIb_C00015435
25	0	1	0			1	2	0	0	1	1	1	0	1	1	1		0	0	0	1	2	2	2	2	2	2	2
75	0	0	1			0	0	0	0	0	0	0	0	0	0	0		0	0	0	0	0	0	0	0	0	0	0
125	2	0	0			2	3	2	2	3	3	2	0	2	2	2		0	0	0	2	2	2	2	2	2	2	2
175	2	0	0			0	3	3	3	3	4	3	0	3	0	3		0	0	0	3	3	3	3	3	3	3	3
225	1	0	0			0	1	1	1	1	1	1	0	1	0	1		0	0	0	2	1	1	1	1	1	1	1
275	2	0	0			2	4	4	4	4	4	4	0	4	1	4		0	0	0	3	3	3	3	3	3	3	3
325	10	0	0			19	3	3	3	3	3	3	0	3	8	3		0	0	0	2	2	2	2	2	2	2	2
375	3	7	0			3	10	10	10	11	10	10	0	10	1	10		0	0	0	10	10	10	10	10	10	10	10
425	3	1	0			2	2	2	2	2	2	2	0	2	0	2		0	0	0	1	1	1	1	1	1	1	1

cdd1

Middle	VIII_C000	XXI_C000	XXXIII_C	XXX_C00	XXXI_C0	XIVa_C0001	XIVb_C0001	Xa_C000	XVII_C00	IXa_C000	IXc_C000	IIIb_CD19	XXII_C00	XXV_C00	IIIc_C000	IIIa_C000	XXXII_C0	0_CD630	0_v_C000	XII_C000	IV_C0001	V_C0001	VI_C0001	VII_C000	XVI_C000	XXVIII_C00015444	XIa_C000	XIb_C00015435
25	1	0	0	11	12	0	0	0	0	0	0	0	0	0	0	0		0	0	0	0	0	0	0	0	0	0	0
75	0	1	0	2	2	0	0	0	0	0	0	0	0	0	0	0		0	0	0	0	0	0	0	0	0	0	0
125	2	2	1	4	4	0	0	1	1	0	0	0	1	0	0	0		0	0	0	1	1	1	1	1	1	1	1
175	0	0	0	2	2	0	0	0	0	0	0	0	0	0	0	0		0	0	0	0	1	1	1	1	1	1	1
225	0	0	0	1	1	0	0	0	0	0	0	0	0	0	0	0		0	0	0	0	0	0	0	0	0	0	0
